# The relationship of cigarette smoking in Japan to lung cancer, COPD, ischemic heart disease and stroke: A systematic review

**DOI:** 10.12688/f1000research.14002.1

**Published:** 2018-02-19

**Authors:** Peter N. Lee, Barbara A. Forey, Alison J. Thornton, Katharine J. Coombs

**Affiliations:** 1 P.N. Lee Statistics and Computing Ltd., Sutton, SM2 5DA, UK; 2 Independent Consultant in Statistics, Dartmouth, TQ6 9HB, UK; 3 Independent Consultant in Statistics, Okehampton, EX20 1SG, UK

**Keywords:** Smoking, lung cancer, COPD, heart disease, stroke

## Abstract

**Background: ** To present up-to-date meta-analyses of evidence from Japan relating smoking to major smoking-related diseases.

**Methods: ** We restricted attention to lung cancer, chronic obstructive pulmonary disease (COPD), ischemic heart disease (IHD) and stroke, considering relative risks (RRs) for current and ex-smokers relative to never smokers.  Evidence by amount smoked and time quit was also considered.  For IHD and stroke only, studies had to provide age-adjusted RRs, with age-specific results considered.  For each disease we extended earlier published databases to include more recent studies.  Meta-analyses were conducted, with random-effects RRs and tests of heterogeneity presented.

**Results:**  Of 40 studies, 26 reported results for lung cancer and 7 to 9 for each other disease.  For current smoking, RRs (95%CIs) were lung cancer 3.59 (3.25-3.96), COPD 3.57 (2.72-4.70), IHD 2.21 (1.96-2.50) and stroke 1.40 (1.25-1.57).  Ex-smoking RRs were lower.  Data for lung cancer and IHD showed a clear tendency for RRs to rise with increasing amount smoked and decrease with increasing time quit.  Dose-response data were unavailable for COPD and unclear for stroke, where the association was weaker.

**Conclusions: ** Compared to studies in other Asian and Western countries, current smoking RRs were quite similar for IHD and stroke.  The comparison is not clear for COPD, where the Japanese data, mainly from cross-sectional studies, is limited.  For lung cancer, the RRs are similar to those in other Asian countries, but substantially lower than in Western countries.  Explanations for this are unclear, but less accurate reporting of smoking by Japanese may contribute to the difference.

## Introduction

It is well established that the relative risk (RR) of lung cancer from smoking is lower in Japan than in Western populations ^1– 5^. However, studies of smoking and lung cancer in Japan have proliferated in recent years, and there have been no meta-analyses in the last 10 years, except for a review of prospective studies reported up to 2008 ^6^. While that review also considered other smoking-related diseases, there have been no other recent comprehensive meta-analyses of the relationship of smoking to chronic obstructive lung disease (COPD) or cardiovascular disease (CVD), based on studies in Japan.

Here we summarize results of Japanese studies relating smoking to lung cancer, COPD, ischaemic (or coronary) heart disease (IHD) and stroke, limiting attention to comparison with never smokers, and considering estimates for current smokers, overall and by amount smoked, and for ex-smokers, overall and by time quit. As we earlier published comprehensive reviews of the evidence for lung cancer ^4^ and COPD ^7^ we extend our meta-analyses to include later papers. For IHD and stroke we extend meta-analyses based on studies published from 1990 ^8^, not attempting to include earlier publications.

Apart from presenting the meta-analysis results, we also briefly compare and contrast the results for Japan with those for other regions, commenting on possible reasons for differences seen.

## Methods

This systematic review was conducted according to PRISMA guidelines ^9^. A completed PRISMA checklist can be found in Supplementary File 1. Throughout this paper we use the RR to include its various estimates, including the odds ratio and the hazard ratio. Where results are referred to as “significant” without further detail, this implies p<0.05.

### Study inclusion and exclusion criteria

We sought studies providing data on RRs for current smokers and/or ex-smokers compared to never smokers for one or more of the diseases lung cancer, COPD, IHD and stroke. Studies providing data from which RRs could be calculated were accepted, as well as those giving the estimates directly.

For lung cancer, attention was restricted to epidemiological prospective or case-control studies involving 100 lung cancers or more, extending our earlier review ^4^ of studies published in the 1900s. That review considered specific histological types of lung cancer, but here we restrict attention to overall lung cancer.

For COPD, cross-sectional studies were also considered, and there was no restriction on number of cases, thus extending an earlier review ^7^ of studies published before 2007. That review also considered chronic bronchitis and emphysema, but we limit attention to studies using the definitions of COPD described there. We also follow the exclusion criteria given in that review.

For IHD and stroke, we extend an earlier review, described in a supplementary file to a recent publication ^8^, which considered studies of prospective or case-control design which involved at least 100 CVD cases and were published between 1990 and 2010. We restricted attention to studies providing data for IHD, acceptable disease definitions including coronary heart disease and acute myocardial infarction, and/or for stroke. Studies providing results only for specific disease subtypes, or only for combined CVD were not included.

While for lung cancer and COPD we accepted studies only providing RRs for the sexes combined, for IHD and stroke the studies had to provide sex-specific RRs. Also for IHD and stroke, studies had to provide RRs adjusted at least for age.

### Literature searches

Studies satisfying the specific criteria were first sought from the three earlier reviews ^4, 7, 8^. Additional papers were also sought from recent reviews of the evidence relating quitting smoking to these diseases ^10– 13^. Finally Medline searches were conducted to update the evidence considered. The searches were conducted along the lines considered in the three earlier reviews ^4, 7, 8^ but restricted to Japan and to a later publication date range – from 2000 for lung cancer, from 2007 for COPD, and from 2010 for IHD and stroke, all searches being conducted in early 2017. No attempt was made to consider studies on IHD and stroke published before 1990. In all the searches, abstracts were first examined, with potentially relevant papers then being obtained and examined in detail.

### Identification of studies

The earlier reviews ^4, 7, 8, 10– 13^ had already allocated relevant papers to studies, noting multiple papers on the same study, and papers reporting on multiple studies. Similar procedures were carried out to continue this process, with some new publications providing updated information on existing studies. As previously, potential overlaps between studies were noted.

### Data recorded

We extended existing databases to include the additional RRs for current or ex-smoking. All estimates considered were for smoking cigarettes only, cigarettes undefined, or any product. The never smoking denominator could include those who never smoked anything, or never smoked cigarettes. RRs were included, where available, for current smoking overall and for sets of RRs grouped by amount smoked, and for ex-smoking overall and for sets of RRs grouped by time quit. As previously, near-equivalent definitions were accepted when exact definitions were not available (e.g. never smokers could include long-term ex-smokers and recent quitters could be treated as current rather than ex-smokers). Given a choice, the RR adjusted for the most potential confounding variables was selected. Sexes-combined RRs were entered only if sex-specific estimates were unavailable. Age-specific RRs were entered, where available, for IHD and stroke, but not for lung cancer or COPD.

### Derivation of RRs

Where necessary RRs were derived from data provided using standard methods, as described elsewhere ^4^.

### Meta-analyses

Fixed-effect and random-effects meta-analyses were calculated using standard methods ^14^ with between estimated heterogeneity quantified by H, the ratio of the heterogeneity chisquared to its degrees of freedom. This is directly related to the I-squared statistic ^15^ by the formula I ^2^ = 100 (H – 1)/H. For all meta-analyses, Egger’s test of publication bias ^16^ was also included. Analyses were conducted for current smoking overall and for ex-smoking overall, preferring cigarette only smoking versus never smoking of anything where there was a choice of definition, and also for about 5, 20 and 45 cigarettes currently smoked and about 12, 7 and 3 years quit for ex-smokers. For an RR to be included in these two dose-response analyses, the grouped level had to include the stated value, but not either of the other two. For IHD and stroke, 20 was replaced by 19 in the above scheme for amount smoked so as to maximise usage of the available data.

For current smoking overall and for ex-smoking overall, meta-analyses were conducted separately by sex with the significance of the between sex difference also estimated. Where sufficient data were available, we also conducted tests of variation by levels of other factors, which varied by disease.

### Study quality and risk of bias

We did not attempt to derive study-specific scores based on study quality and risk of bias, as the relative importance of different sources of bias or poor quality cannot be reliably assessed. Instead we carried out some meta-analyses showing how estimates varied by aspects of study quality and bias, including study size, number of adjustment variables, and study type. We also considered factors affecting quality and bias in the discussion section.

## Results

### Searches

For lung cancer, the earlier review ^4^ included 19 studies conducted in Japan, of which 11 provided relevant data. Three additional relevant studies were reported in the quitting review ^11^ with a further 12 found from the updated Medline search.

For COPD, the earlier review ^7^ included four studies in Japan, one rejected as having no relevant data and the results from another later being found to be superseded by a more recent publication. No additional studies were identified in the quitting review ^13^ but the updated Medline search found four further relevant studies.

For IHD and stroke, the earlier review ^8^ included four relevant publications, three describing individual studies and one a pooled analysis of three studies. One additional study was identified in the quitting reviews ^10, 12^, and four additional relevant publications were identified from the Medline search, three describing individual studies and one a pooled analysis of ten studies.

Supplementary File 2 provides fuller details of the literature searches.

### Studies identified

Table 1 gives details of the 40 studies included in the analyses, presented in order of the date of the publication reporting the relevant results. Of these, the numbers giving results for lung cancer, COPD, IHD and stroke are, respectively, 26, seven, nine and seven, some studies reporting on more than one of these diseases. The table provides information for each study on the study type, the location, the years in which it was conducted, the population considered, and the number of cases of each disease it considered. Two publications based on the Japan Collaborative Cohort (JACC) are treated as separate studies in the table as the publications relate to different diseases and periods of follow-up. The same is true for three publications based on the Japan Public Health Center (JPHC) study.

**Table 1.** Details of studies included.

Study ref	Year of Publ.	Study type ^a^	Location in Japan	Years ^b^	Population ^c^	Number of cases ^d^			
						LC	COPD	IHD	Stroke
HITOSU ^17^	1968	CC	Amagaski, Nishinomiya	1960–1966	Aged 35–74	216			
SEGI2 ^18^	1979	CC	Tokyo, Sendai	1962–1970	Any age	378			
TSUGAN ^19^	1987	CC	National	1976–1985	Aged 30–49	185			
SOBUE2 ^20^	1988	CC	Osaka	1965–1983	Aged 20+	2083			
HIRAYA ^21^	1990	P	Six prefectures	1965/1982	Aged 40+	1917		3548	12732
YAMAGU ^22^	1992	CC	Kitakyushu	1989–1990	Any age	144			
GAO2 ^23^	1993	CC	Tokai area	1984–1986	Aged 30–84	282			
SOBUE ^24^	1994	CC	Osaka	1986–1991	Any age	1376			
KIHARA ^25^	1995	CC	Kanagawa	1991–1993	Any age	447			
WAKAI ^26^	1997	CC	Okinawa	1988–1991	Aged 40–89	333			
STELL2 ^3^	2001	CC	Nagoya	1993–1998	Aged 20–81	410			
JPHC(SOBUE) ^1^	2002	P	National	1990–1994/1999	Aged 40–69	422			
HIRAKI ^27^	2003	CC	Aichi	1991–2000	Aged 26–81	192			
KAWAMI ^28^	2003	P	National	1980–1999	Aged 30+	106			
MINAMI ^29^	2003	CC	Miyagi	1997–2001	Aged 40	515			
YAMAGI ^30^	2003	P	Ikawa, Kyowa, Yao City	1975/1986	Men aged 40–69			100	257
FUKUCH ^31^	2004	CS	18 prefectures	2000	Aged 40+		256		
MARUG2 ^32^	2004	CC	Osaka, Okinawa, Nagano	1996–1998	Aged 40–79	1115			
UESHIM ^33^	2004	P	National	1980/1994	Aged 30+			69	203
KANASH ^34^	2005	CC	Ibaraki	1997–2003	Men aged 50–79	363			
MARUG1 ^35^	2005	P	Three prefectures	1983–1990/2000	Aged 40–80	598			
TSUSHI ^36^	2006	CS	Azumi, Kouhoku, Nagano	2003–2004	Mean age 54		48		
JACC(OZASA) ^37^	2007	P	45 areas	1988–1990/2003	Aged 40–79	1087			
KOJIM2 ^38^	2007	CS	Tokyo	1997–2005	Aged 25–74		466		
OSAKI ^39^	2007	P	Tottori	1995/1999	Men of any age	119			
HIRAY2 ^40^	2009	CC	Aichi, Gifu, Kyoto	2006	Aged 56–75		278		
3 STUDIES ^41, 42^	2010	P	National ^e^	1983–1993/2003	Aged 40–79	968		493	1472
KIYOHA ^43^	2010	CC	Kyushu	1996–2008	Any age	462			
OSAKA ^44^	2010	CS	Takahata	2004–2005	Aged 40+		308		
JPHC(SHIMAZU) ^45^	2010	P	National	1995–1999/2005	Aged 45–74	481			
KONDO ^46^	2011	P	Seven workplaces	2000–2008/2008	Male workers aged 20–61			37	73
OMORI2 ^47^	2011	CS	Kumamoto	1994–1999	Males aged 30–76		91		
AKIBA ^48^	2012	P	Hiroshima, Nagasaki	1958/1999	Atomic bomb survivors – any age	610			
10 STUDIES ^49^	2012	P	National ^f^	1977–1997/ Varies	Aged 40–89			382	893
HORIE ^50^	2013	CS	Tokyo	2001–2008	Aged 30+		1035		
ITO ^51^	2013	CC	Aichi	2001–2005	Any age	1552			
JPHC(ESHAK) ^52^	2014	P	National	1990–1993/2009	Aged 45–74			584	
FUKUMO ^53^	2015	CC	Aichi	1993–1998	Aged 20–81	625			
HATANA ^54^	2015	P	National	2003/2011	Men aged 30–55 in health insurance program			238	
JACC(MATSUNAGA) ^55^	2017	P	45 areas	1988–1990/2009	Aged 40–79			1554	3163

^a^ C = case-control study, CS = cross sectional study, P = prospective study

^b^ * = unknown. Values in brackets are approximate, based on one year before the first publication. For prospective studies, baseline year(s)/final follow-up year.

^c^ Unless shown otherwise in this column, the study specified no major inclusion or exclusion criteria.

^d^ In whole study.

^e^ Combined results from three prospective studies; the JPHC and JACC studies had wide national coverage, and the TPCS was conducted in three prefectures (Miyagi, Aichi, Osaka). The first reference cited gives the lung cancer findings and the second the cardiovascular findings.

^f^ Combined results from ten prospective studies, each with at least 10 years follow-up. These included the JACC study.

Two pooled analyses of results are treated as single studies in Table 1. The pooled analysis of three studies reported by Wakai *et al* ^41^ for lung cancer and by Honjo *et al* ^42^ for CVD included results from the JACC, JPHC and MARUG1 studies. It may have some overlap of results for lung cancer with the findings from JACC (OZASA), JPHC (SOBUE and SHIMAZU) and MARUG1 and for CVD with the findings from JACC (MATSUNAGA) and JPHC (ESHAK). The pooled analysis of ten studies on CVD by Nakamura *et al* ^49^ may have some overlap of results with the findings from UESHIM and JACC (MATSUNAGA), but is predominantly based on studies not considered elsewhere.

Of the 40 studies, 18 are case-control and 6 of cross-sectional design (all of COPD), with the rest prospective. Ten of the studies were published before 2000, although 20 had been completed by then. The largest study was HIRAYA, which involved 1917 lung cancer cases, 3,548 cases of IHD and 12,732 of stroke, though the SOBUE2 study involved somewhat more lung cancer cases, 2,083.

### Relative risks included

Table 2 gives the RRs for current and ex-smoking while Supplementary File 3 gives them by amount smoked and time quit. As seen in Table 2, most lung cancer estimates are adjusted for age plus at most one other potential confounding variable, while nearly all COPD estimates are unadjusted, even for age. All the IHD and stroke estimates are (as required) adjusted for age, and most of these also for a number of additional variables.

**Table 2.** Relative risks for current and for ex-smokers (vs never smokers).

Disease	Study ref	Sex	Age	Relative risks (95%CI)		Adjustment factors
				Current smoker	Ex-smoker	
Lung cancer	HITOSU	M		2.79 (1.27–6.09)	3.95 (1.63–9.55)	Age
		F		3.09 (1.82–5.27)	6.72 (2.55–17.68)	Age
	SEGI2	M		3.74 (1.75–8.00)	-	Age
		F		1.65 (0.90–3.02)	-	Age
	TSUGAN	M		1.22 (0.60–2.50)	1.53 (0.50–4.68)	None
	SOBUE2	M		4.47 (3.89–5.14)	-	Age +1
		F		3.28 (2.79–3.87)	-	Age +1
	HIRAYA	M		4.45 (3.60–5.50)	1.71 (1.08–2.72)	Age
		F		2.34 (1.87–2.92)	2.98 (1.14–7.77)	Age
	YAMAGU	M+F		4.90 (2.55–9.44)	2.90 (1.43–5.90)	Age
	GAO2	M		6.61 (3.47–12.58)	3.56 (1.83–6.91)	Age
	SOBUE	M		4.10 (2.80–5.90)	2.80 (1.90–4.20)	Age
		F		2.80 (2.00–3.90)	2.10 (1.40–3.20)	Age
	KIHARA	M+F		4.06 (3.00–5.49)	1.83 (1.20–2.79)	None
	WAKAI	M		4.40 (2.19–8.85)	2.43 (1.16–5.06)	Age +1
		F		4.37 (2.21–8.62)	5.33 (1.21–23.50)	Age +1
	STELL2	M		6.30 (3.70–10.90)	2.20 (1.30–4.00)	Age +1
	JPHC(SOBUE)	M		4.50 (3.00–6.80)	2.20 (1.40–3.40)	Age +1
		F		4.20 (2.40–7.20)	3.70 (1.40–10.20)	Age +1
	HIRAK1	M+F		2.11 (1.35–3.31)	1.65 (0.99–2.75)	1 (not age)
	KAWAMI	M		6.76 (2.13–21.48)	2.35 (0.62–8.91)	Age
		F		3.67 (1.55–8.68)	-	Age
	MINAMI	M		4.75 (3.04–7.42)	2.74 (1.71–4.38)	Age +4
		F		1.91 (1.14–3.18)	2.37 (1.08–5.23)	Age +4
	MARUG2	M		2.78 (1.94–4.00)	2.46 (1.47–4.12)	Age +1
		F		2.34 (1.46–3.74)	0.93 (0.47–1.81)	Age +1
	KANASH	M		6.31 (3.33–11.97)	2.97 (1.55–5.70)	Age +1
	MARUG1	M		5.10 (3.34–7.79)	2.60 (1.65–4.10)	Age +1
		F		3.66 (2.50–5.35)	2.94 (1.63–5.31)	Age +1
	JACC(OZASA)	M		4.94 (3.77–6.47)	2.20 (1.63–2.96)	Age +1
		F		4.25 (2.98–6.05)	2.19 (1.07–4.48)	Age +1
	OSAKI	M		4.90 (2.80–8.40)	2.20 (1.20–4.10)	Age
	3 STUDIES	M		4.71 (3.76–5.89)	2.10 (1.66–2.67)	Age +1
	KIYOHA	M+F		2.10 (1.55–2.84)	3.70 (2.44–5.60)	None
	JPHC(SHIMAZU)	M		3.29 (2.55–4.24)	2.53 (1.85–3.45)	1 (not age)
	AKIBA ^a^	M	Any	3.19 (2.27–4.47)	2.50 (1.50–4.30)	1 (not age), age +4
		F	Any	3.14 (2.55–3.88)	1.40 (0.70–2.60)	1 (not age), age +4
	ITO	M+F		4.34 (3.47–5.44)	2.02 (1.60–2.55)	Age +4
	FUKUMO	M+F		3.40 (2.71–4.27)	1.34 (1.02–1.75)	None
COPD	FUKUCH	M+F		2.96 (2.14–4.09)	2.99 (2.12–4.22)	None
	TSUSHI	M+F		5.79 (2.51–13.38)	4.81 (1.93–12.00)	None
	KOJIM2	M		2.55 (1.88–3.46)	1.68 (1.20–2.34)	None
		F		1.11 (0.45–2.77)	0.18 (0.01–2.92)	None
	HIRAY2	M		21.31 (6.35–71.48)	35.81 (11.06–115.94)	None
		F		56.70 (9.54–337.02)	84.00 (18.42–382.98)	None
	OSAKA	M+F		2.74 (2.16–3.48)	2.48 (1.89–3.25)	None
	OMORI2	M		4.73 (2.36–9.46)	2.39 (1.24–4.59)	Age +1
	HORIE	M		3.59 (2.98–4.33)	2.00 (1.66–2.40)	Age
		F		3.68 (2.27–5.96)	0.77 (0.31–1.91)	Age
IHD	HIRAYA	M	40+	1.73 (1.52–1.97)	1.39 (1.06–1.83)	Age
		F	40+	1.90 (1.66–2.18)	0.73 (0.24–2.24)	Age
	YAMAGI	M	40–69	4.39 (1.57–12.24)	3.70 (1.20–11.20)	Age +11
	UESHIM	M	30+	2.14 (0.77–5.91)	1.00 (0.28–3.53)	Age +5
		F	30+	1.24 (0.33–4.65)	0.87 (0.11–6.70)	Age
	3 STUDIES	M	40–64	2.50 (1.88–3.34)	1.78 (1.28–2.46)	Age +1
		M	65–79	1.92 (1.46–2.53)	1.68 (1.26–2.24)	Age +1
		F	40–64	4.36 (3.01–6.32)	2.79 (1.30–6.00)	Age +1
		F	65–79	2.21 (1.62–3.02)	2.22 (1.44–3.40)	Age +1
	KONDO	M	20–61	4.76 (1.40–16.25)	0.83 (0.15–4.50)	Age +3
	10 STUDIES	M	40–64	2.25 (1.21–4.21)	0.83 (0.34–1.98)	Age +4
		M	65–89	2.01 (1.26–3.22)	1.11 (0.63–1.96)	Age +4
		F	40–64	3.52 (1.61–7.68)	4.25 (1.01–17.94)	Age +4
		F	65–89	2.89 (1.73–4.83)	1.90 (0.77–4.71)	Age +4
	JPHC	M	45–74	2.26 (1.79–2.87)	1.09 (0.83–1.43)	Age +14
		F	45–74	2.89 (1.94–4.30)	No cases in ex-smokers	Age +14
	HATANA	M	30–39	2.17 (1.08–4.34)	-	Age +8
		M	40–55	1.34 (1.01–1.79)	-	Age +8
	JACC	M	40–79	1.95 (1.58–2.39)	1.29 (1.02–1.63)	Age +6
		F	40–79	2.45 (1.89–3.18)	1.07 (0.58–1.95)	Age +8
Stroke	HIRAYA	M	40+	1.08 (1.02–1.14)	0.99 (0.87–1.13)	Age
		F	40+	1.18 (1.10–1.28)	1.53 (1.08–2.15)	Age
	YAMAGI	M	40–69	1.41 (0.97–2.06)	0.80 (0.50–1.30)	Age +11
	UESHIM	M	30+	1.69 (0.98–2.93)	1.56 (0.84–2.90)	Age +5
		F	30+	1.66 (0.91–3.03)	1.31 (0.50–3.39)	Age +5
	3 STUDIES	M	40–64	1.41 (1.16–1.71)	0.97 (0.77–1.22)	Age +1
		M	65–79	1.13 (0.95–1.33)	1.02 (0.85–1.21)	Age +1
		F	40–64	2.75 (2.15–3.53)	1.85 (1.10–3.10)	Age +1
		F	65–79	1.24 (0.99–1.56)	1.09 (0.77–1.53)	Age +1
	KONDO	M	20–61	2.17 (1.09–4.30)	1.00 (0.42–2.41)	Age +3
	10 STUDIES	M	40–64	2.58 (1.54–4.33)	1.40 (0.72–2.71)	Age +4
		M	65–89	1.36 (1.02–1.81)	1.02 (0.73–1.43)	Age +4
		F	40–64	1.79 (0.98–3.26)	2.11 (0.67–6.68)	Age +4
		F	65–89	1.17 (0.75–1.83)	1.25 (0.64–2.43)	Age +4
	JACC	M	40–79	1.23 (1.07–1.42)	0.91 (0.78–1.06)	Age +6
		F	40–79	1.35 (1.08–1.68)	0.97 (0.66–1.43)	Age +8

^a^Ex-smoker data are from an earlier reference ^56^

### Meta-analyses

Meta-analysis results (random effects estimates) are shown for current smoking in Table 3, for amount smoked by current smokers in Table 4, for ex-smoking in Table 5 and for time quit by ex-smokers in Table 6. Supplementary File 4 gives some additional results for current and ex-smoking for lung cancer, IHD and stroke. Below we summarize the results by disease risk.

**Table 3.** Meta-analyses for current smoking.

Characteristic	Level	Statistic ^a^	Lung cancer	COPD	IHD	Stroke
All	All	n	39	10	20	16
		R	3.59 (3.25–3.96)	3.57 (2.72–4.70)	2.21 (1.96–2.50)	1.40 (1.25–1.57)
		H, P _H_	2.98, p<0.001	3.71, p<0.001	2.53, p<0.001	5.21, p<0.001
Sex	Male	n	20	4	12	9
		R	4.20 (3.74–4.72)	4.07 (2.59–6.40)	1.98 (1.74–2.25)	1.32 (1.16–1.51)
	Female	n	13	3	8	7
		R	3.00 (2.61–3.44)	4.90 (1.08–22.26)	2.59 (2.06–3.27)	1.50 (1.16–1.94)
	Combined	n	6	3	0	0
		R	3.27 (2.51–4.28)	3.00 (2.34–3.85)	-	-
	Between levels	P _B_	<0.001	NS	<0.05	<0.01

^a^ n = number of estimates combined, R = random-effects meta-analysis RR (95% CI), H = heterogeneity chisquared per degree of freedom, P _H_ = probability value for heterogeneity expressed as p<0.001, p<0.01, p<0.05, p<0.1 or NS (p≥0.1). P _B_ = probability value for between level comparison similarly expressed.

**Table 4.** Meta-analysis for amount smoked by current smokers.

Amount smoked	Statistic ^a^	Lung Cancer	COPD	IHD	Stroke
Number of sets ^b^		21	0	12	8
About 5 cigs/day ^c^	n	16	-	5	5
	R	2.89 (2.44–3.43)		1.71 (1.50–1.94)	1.38 (1.15–1.65)
About 20 cigs/day ^c^	n	12	-	5	5
	R	4.43 (3.68–5.34)		1.91 (1.55–2.35)	1.29 (1.07–1.56)
About 45 cigs/day ^c^	n	16	-	11	8
	R	6.42 (5.14–8.02)		2.70 (2.16–3.39)	1.64 (1.21–2.22)

^a^ n = Number of estimates combined, R = random effects meta-analysis RR (95% CI).

^b^ Number of sets of RRs available for the key value analyses, where the dose for comparison is never smoked. See also Supplementary File 3 for details.

^c^ Base for comparison is never smoked. For lung cancer and COPD; the first category for which results are provided includes 5 cigs/day, but does not include 20 cigs/day; the second includes 20 cigs/day, but does not include 5 or 45 cigs/day; and the third includes 45 cigs/day, but does not include 20 cigs/day. For IHD and stroke; 20 cigs/day is replaced by 19.

**Table 5.** Meta-analyses for ex-smoking.

Characteristic	Level	Statistic ^a^	Lung cancer	COPD	IHD	Stroke
All	All	n	34	10	17	16
		R	2.26 (2.03–2.52)	3.03 (2.00–4.57)	1.46 (1.24–1.71)	1.05 (0.96–1.15)
		H, P _H_	1.50, <0.05	6.89, <0.001	1.58, <0.1	1.26, NS
Sex	Male	n	18	4	10	9
		R	2.36 (2.12–2.63)	3.04 (1.65–5.62)	1.37 (1.18–1.61)	0.98 (0.91–1.06)
	Female	n	10	3	7	7
		R	2.35 (1.70–3.25)	2.50 (0.07–85.77)	1.75 (1.17–2.60)	1.29 (1.06–1.55)
	Combined	n	6	2	0	0
		R	2.04 (1.51–2.75)	2.77 (2.21–3.48)	-	-
	Between levels	P _B_	NS	<0.1	<0.01	<0.01

^a^ n = number of estimates combined, R = random-effects meta-analysis RR (95% CI), H = heterogeneity chisquared per degree of freedom, P _H_ = probability value for heterogeneity expressed as p<0.001, p<0.01, p<0.05, p<0.1 or NS (p≥0.1). P _B_ = probability value for between level comparison similarly expressed.

**Table 6.** Meta-analysis for duration of quitting.

Duration of quitting	Statistic ^a^	Lung Cancer	COPD	IHD	Stroke
Number of sets ^b^		11	1 ^c^	4	4
About 12 years ^d^	n	9	-	4	4
	R	2.08 (1.65–2.61)		1.22 (0.81–1.84)	0.95 (0.72–1.23)
About 7 years ^d^	n	8	-	4	4
	R	2.87 (2.32–3.56)		2.08 (1.48–2.90)	1.13 (0.85–1.50)
About 3 years ^d^	n	9	-	4	4
	R	3.70 (3.12–4.40)		2.10 (1.20–3.69)	1.28 (1.11–1.48)

^a^ n = Number of estimates combined, R = random effects meta-analysis RR (95% CI).

^b^ Number of sets of RRs available for the key value analyses, where the comparison is with never smoked. See also Supplementary File 3 for details.

^c^ One study reported RRs (95% CIs) of 2.08 (1.08–4.00) for "early quitters" and 2.42 (1.11–5.25) for "late quitters", early quitters having reported current smoking in 1994 but not in 1999 or 2006, and late quitters having reported current smoking in 1994 and 1999 but not in 2006.

^d^ Base for comparison is never smoked. The first category for which results are provided includes quit 12 years ago but does not include quit 7 years ago; the second includes quit 7 years ago but does not include quit 3 or 12 years ago; and the third includes quit 3 years ago but does not include quit 7 years ago.

### Lung cancer

For current smoking, the overall RR shown in Table 3 is 3.59 (95%CI 3.25–3.96), based on 39 estimates. As shown in Table 2, the RRs range from 1.22 to 6.76, with all but two statistically significant. While the estimates are heterogeneous, no single factor is responsible for this, though (see also Supplementary File 4) there is evidence that RRs are higher in males and where adjusted for more variables. Table 4 shows that the RRs increase steadily with amount smoked, rising from 2.89 (2.44–3.43) for “about 5 cigs/day” to 6.42 (5.14–8.02) for “about 45 cigs/day”.

Compared to current smoking the RR for ex-smoking ( Table 5) of 2.26 (2.03–2.52) is lower and shows less heterogeneity, with the only factor showing significant variation being publication year, with RRs higher in older (pre-1980) studies. RRs clearly reduced with increasing time of quit, evident both in the individual data sets for each study and the summary analysis in Table 6.

### COPD

For current smoking, the overall RR shown in Table 3 is 3.57 (95%CI 2.72–4.70), based on 10 estimates. However, as Table 2 shows, the two RRs from HIRAY2 are atypically high, and excluding these results substantially reduced the heterogeneity and reduced the RR to 3.10 (2.57–3.75). There is no significant variation by sex. Analyses by further subgroups were not attempted, due to the limited number of estimates for which results are available. There are no available results by amount smoked.

For ex-smoking (see Table 5) the overall RR was 3.03 (2.00–4.57). This reduced to 2.16 (1.68–2.77) after excluding the high results from HIRAY2 ( Table 2). A single study reported similar RRs for late quitters and early quitters, as shown in the footnote to Table 6.

### IHD

For current smoking, the overall RR ( Table 3) is 2.21 (95%CI 1.96–2.50). However, the estimates are clearly heterogeneous, with RRs somewhat higher in females than males. The additional results in Supplementary File 4 show that RRs tend to be greater in more recently published studies, but did not vary significantly by age or by the number of variables adjusted for. There was variation by study size, but this did not show any systematic trend. As shown in Table 4, the RRs increase steadily with amount smoked, rising from 1.71 (1.50–1.94) for “about 5 cigs/day” to 2.70 (2.16–3.39) for “about 45 cigs/day”.

For ex-smoking, the overall RR ( Table 5) of 1.46 (1.24–1.71) is clearly lower than that for current smoking. RRs tended to be higher in females, and in less adjusted estimates. In those who had quit for “about 12 years” the RR at 1.22 (0.81–1.84) is not significantly increased, but those for shorter quit times are both elevated to a similar extent ( Table 6).

### Stroke

For current smoking, the overall RR ( Table 3) is 1.40 (95%CI 1.25–1.57), less elevated for the other diseases. There is clear heterogeneity, RRs tending to be higher in females, for those aged <65, in more recently published studies, and in studies involving fewer cases (see also Supplementary File 4).

There is no clear relationship of risk of stroke to amount smoked ( Table 4) though the largest estimate is for the highest dose.

Overall, risk is not significantly elevated in former smokers ( Table 5) with the RR estimated as 1.05 (0.96–1.15). However, the analyses show some increase in females, and in short-term quitters ( Table 6).

### Publication bias

Each meta-analysis included a test of publication bias (detailed results not shown).

For lung cancer, there is no evidence of publication bias for current smoking and only marginal evidence (p<0.05) for ex-smoking, where RRs were somewhat greater in smaller studies. For CVD, the strongest evidence of publication bias (p = 0.003) is for the analysis of current smoking RRs for stroke. This corresponds with the evidence of higher risks in smaller studies. No evidence of publication bias is seen for COPD for either current or ex smoking.

### Avoiding overlap

The meta-analyses reported include all available data, accepting some overlap of results between studies. Some additional analyses were conducted for current smoking either omitting results from the publications reporting combined analyses (3 STUDIES, 10 STUDIES) or omitting results from studies considered in these analyses (JPHC, JACC, MARUG1). For lung cancer, compared to the original estimate of 3.59 (95%CI 3.25–3.96), the first omission gave 3.55 (3.20–3.93) while the second gave 3.46 (3.06–3.91). For IHD, the original RR of 2.21 (1.96–2.50) became 2.01 (1.76–2.29) for the first omission and 2.20 (1.88–2.57) for the second. For stroke 1.40 (1.25–1.57) became 1.22 (1.11–1.34) and 1.44 (1.25–1.65). For CVD it should be noted that omitting the 10 STUDIES results lost relevant information as many of its individual studies were not included elsewhere.

## Discussion

The results presented show some increased risk of all four diseases with current smoking, and a lesser increase with ex-smoking, that with stroke not being clearly significant. The evidence for COPD is clearly the thinnest being based largely on cross-sectional studies and on unadjusted RRs, and providing little or no data for amount smoked or time quit. The other diseases do show a tendency for RRs to increase with amount smoked and to decline with increasing time quit, though again the associations are less clear for stroke, the disease most weakly associated with smoking.

In considering these results, various aspects of the data require comment.

### Product used

Smoking of tobacco products other than cigarettes, such as cigars or pipes, is rare in Japan ^57^ and whether authors related results to unspecified smoking, to cigarette smoking or to cigarette only smoking would be of little practical relevance. Similarly the precise definition of the comparison group, never smokers, is unlikely to be important.

### Study type

For lung cancer, there is no evidence that RRs differ between prospective and case-control studies. Since all the RRs for CVD came from prospective studies, and virtually all those for COPD came from cross-sectional studies, variation by study type could not usefully be examined for these diseases.

### Subtypes of disease

It was beyond the scope of the study to investigate variation by disease subtypes, though we note that, for lung cancer, some studies (e.g. AKIBA, ITO, MARUG2, JPHC(SOBUE)) present evidence consistent with there being higher RRs for squamous cell carcinoma than for adenocarcinoma.

### Age-specific results

It has previously been established that the variation in RR by age is much greater for cardiovascular disease than for lung cancer or COPD ^4, 7, 8^. For this reason we only considered age-specific data for IHD and stroke. The results generally confirmed the higher RRs in younger individuals.

### Adjustment for potential confounding variables

In order to limit the scope of the project, attention was restricted to RRs adjusted for the most potential confounding variables where there was a choice. For lung cancer, there was some evidence that more adjusted RRs were higher, but for cardiovascular disease no such trend was seen. RRs for COPD were generally unadjusted.

### Outliers

Formal tests for outliers were not attempted, but it was evident from inspection of Table 2 that the very large RRs for the HIRAY2 study were inconsistent with the rest of the available results, and removal of the results from the meta-analysis materially reduced the RRs for both current and ex-smoking. Otherwise there seemed to be no clear outliers, unusually low or high RRs typically having a very wide 95%CI, being based on limited data.

### Other issues

Imprecision of the effect estimates could have resulted from errors in diagnosis of disease or errors in determining smoking habits. It was notable that mortality studies generally did not rely on autopsy-confirmed diagnosis, and that smoking habits recorded were usually based on self-report by the individual with no confirmation of non-smoking status by measurement of biomarkers such as cotinine.

### Comparison with results for Western populations

Table 7 presents meta-analysis relative risks for current smoking by region from this study, from reviews of ours ^4, 7, 8^ and from other selected recent reviews ^2, 6, 58– 60^ chosen as they provided RR estimates for the sexes combined by region. It was clear for IHD that there is little evidence of a material difference in RR between estimates from Japanese studies and those from studies in other Asian countries or Western countries. In all cases the RR is quite close to 2. The pattern is broadly similar for stroke, with the RR for stroke, typically about 1.4, less than that for IHD, with the minor exception of Scandinavia, where the RR is based on only two estimates. For COPD, the available data are limited, but provide some suggestion that, compared to Japan, RRs are somewhat higher for North America though similar for Europe.

Evidence of international variation in current smoking RRs is much clearer for lung cancer, where the meta-analysis RRs reported for Japan and other Asian countries range from 2.46 to 3.64, while those for North America, Europe and Australia/New Zealand are substantially higher, ranging from 7.53 to 12.55. The explanation for this difference has been discussed in a number of previous publications (e.g. 1– 3, 35) without any clear explanation being offered. An international case-control study involving populations in the USA and Japan ^3^ found no substantial international differences in average daily consumption or mean duration of smoking, but noted that US cases began smoking 2.5 years earlier than Japanese cases. They suggested that possible explanations for the higher smoking risk in the US study may “include a more toxic cigarette formulation of American manufactured cigarettes as evidenced by higher concentrations of tobacco-specific nitrosamines in both tobacco and mainstream smoke, the much wider use of activated charcoal in the filters of Japanese than in American cigarettes, as well as documented differences in genetic susceptibility and lifestyle factors other than smoking.” Other authors ^1, 2, 35^ have referred to the severe shortage of cigarettes in Japan during and shortly after World War II, the higher incidence of lung cancer in nonsmokers in Japan due to indoor air pollutants (including environmental tobacco smoke), the low fat intake and high intake of several phytochemicals in the Japanese diet, and the lower indoor radon concentrations in Japan than in the USA.

**Table 7.** Current smoking relative risks in the present study compared with those reported in other studies in Japan and elsewhere.

Disease	Source	Region	N	RR (95%CI)
Lung cancer	This review	Japan	39	3.59 (3.25–3.96)
	Lee *et al*. 2012 ^4^	N America	84	11.68 (10.61–12.85)
		UK	25	7.53 (5.40–10.50)
		Scandinavia	21	8.68 (7.14–10.54)
		Other Europe	23	8.65 (5.98–12.51)
		China	5	2.94 (2.23–3.88)
		Japan	18	3.55 (3.05–4.14)
		Other Asia	7	2.90 (2.04–4.13)
	Wakai *et al*. 2006 ^2^	Japan	23	3.64 (3.34–3.97) ^a^
	Nakamura *et al*. 2009 ^6^	Asia	NA	3.54 (3.00–4.17)
	Huxley *et al*. 2007 ^58^	Asia	NA	2.46 (2.00–3.04) ^a^
		Australia/NZ	NA	12.55 (8.47–18.60) ^a^
COPD	This review	Japan	10	3.57 (2.72–4.70)
		(omitting outliers)	8	3.10 (2.57–3.75)
	Forey *et al*. 2011 ^7^	N America	39	4.56 (3.69–5.62)
		Europe	55	3.31 (2.80–3.92)
		Asia	17	2.86 (2.27–3.60)
	Nakamura *et al*. ^6^	Asia	NA	1.40 (1.18–1.66)
IHD	This review	Japan	20	2.21 (1.96–2.50)
	Lee *et al*. 2017 ^8^	N America	61	1.94 (1.77–2.12)
		W Europe	4	2.24 (1.49–3.39)
		Scandinavia	10	2.46 (1.85–3.37)
		Japan	9	2.21 (1.85–2.65)
		Other Asia	8	2.15 (1.56–2.96)
	Nakamura *et al*. 2009 ^6^	Asia	NA	1.97 (1.66–2.23)
	Asia Pacific Cohort Studies Collaboration ^59^	Asia	NA	1.75 (1.60–1.90)
Stroke	This review	Japan	16	1.40 (1.25–1.57)
	Lee *et al*. 2017 ^8^	N America	33	1.50 (1.31–1.71)
		W Europe	4	1.49 (1.18–1.89)
		Scandinavia	2	2.72 (1.82–4.07)
		Japan	9	1.37 (1.19–1.58)
		Other Asia	9	1.33 (1.18–1.51)
	Nakamura *et al*. 2009 ^6^	Asia	NA	1.34 (1.21–1.48)
	Asia Pacific Cohort Studies Collaboration ^59^	Asia	NA	1.43 (1.32–1.54)
	Wang *et al*. 2008 ^60^	China	NA	1.22 (1.08–1.37)

^a^ Estimated from data for sexes separately

Whether lung cancer risk in nonsmokers in Japan is higher than in Western countries is in any case open to question. A recent publication ^61^ that indirectly estimated absolute lung cancer mortality rates by smoking status based on a systematic review, found that they were quite similar in Japan to those in most Western countries. For age 70–74 years, mortality rates (per 100,000 per year) in those who had never smoked were estimated as 42.5 (95% CI 34.5–52.4) in Japan based on n = 14 estimates, as compared, for example, to 37.6 (32.6–43.3, n = 54) for the USA, 61.5 (46.8–80.8, n = 26) for the UK, 29.6 (21.9–40.0, n = 20) for Scandinavia, 38.2 (29.3–49.8, n = 31) in other countries in Western Europe, and 32.3 (22.3–46.8, n = 11) for Eastern Europe. It was China, not Japan, that had a markedly higher lung cancer rate of 99.1 (90.2–108.8, n = 38) in never smokers.

One potential explanation for the difference in the relative risk of lung cancer between Asian and Western populations may lie in differences in the accuracy of reporting smoking habits. We are currently involved in a separate project to review accuracy of reporting smoking habits, using cotinine to validate self-reported smoking habits. We are aware of five studies in Asian populations, three in Japan ^62– 64^, one in Korea ^65^ and one of South-East Asians resident in the USA ^66^, which report results separately for never, ex and current smokers and by sex. All five give results for women, and four do so for men, and the proportion of true current smokers in self-reported never or ex-smokers (as judged by high cotinine levels) in women (range 12.3% to 61.6%, overall 45.8%) is much higher than it is men (range 0.4% to 6.0%, overall 3.4%). The proportion is also much higher than in 13 data sets (five in males, five in females, three in sexes combined) reported in six publications ^67– 72^ describing studies in Western populations (England, Finland, Germany, USA) involving large numbers (>2000) of subjects. Here percentages range from 0.4% to 6.1%, with the overall estimates 1.6% for males, 3.2% for females, and 2.3% for the whole sample.

Although the difference is impressive, the percentage that affects the relative risk for current versus never smokers is the proportion of the current smokers in self-reported never smokers. Here the overall percentages are 7.8% in Asian females, 5.5% in Asian males, 1.4% in Western females and 2.3% in Western males. If one assumes that the true RR for current smoking and lung cancer is X, the observed RR based on self-reported data will be X / (1 + (X − 1) p) where p is the proportion of true current smokers among self-reported never smokers. Thus if X = 10, the observed RRs would be 5.9 in Asian females, 6.7 in Asian males, 8.9 in Western females and 8.3 in Western males, based on the data sets investigated. Although there are difficulties in interpreting these results for various reasons, including between-study variation in the body fluids and cut-offs used to determine true smokers, and the possibility that self-reported never smokers who are considered to be current smokers may smoke less than current smokers who admit smoking, we feel that these results suggest that different levels of misclassification of smoking habits between Asian and Western populations may contribute to the lower observed current smoker RRs in Asian populations.

### Passive smoking

This review is concerned with the effects of active smoking in Japan on the four diseases concerned. Recent reviews by ourselves ^73– 76^ and others ^77^ have found that evidence in Japan on passive smoking is very sparse, except for lung cancer. For IHD, our recent review ^75^ cites only the Hirayama study ^78^ as reporting a non-significant relative risk of 1.16 (95% CI 0.94–1.43), while our review of passive smoking and stroke ^74^ cites the Hirayama study as finding “no significant trend” and a study by Nishino *et al* ^79^ as giving a relative risk of 0.75 (0.80–1.12). Our review of passive smoking and COPD ^76^ again cited only the relative risk from the Hirayama study of 1.38 (0.86–2.21), though one very recently published study by Ukawa *et al* ^80^ did report significantly increased RRs of 2.40 (1.39–4.15) and 2.88 (1.68–4.93) for passive smoking at home for ≤4 days per week and almost every day, as compared to none.

For lung cancer, the evidence is much more extensive and two recent reviews ^73, 77^ reported very similar overall relative risks for spousal or at home smoking of 1.26 (1.11–1.45) and 1.28 (1.10–1.48) based on 13 or 12 individual estimates, although our review ^73^ suggested that most, if not all, of the ETS/lung cancer association might be explained by inadequate adjustment for potential confounding by diet and education and by bias due to misclassification of some true smokers as nonsmokers. Even were this association a causal result of exposure to passive smoking it could not explain the substantial difference in active smoking RRs between Asian and Western populations. Not only do the RRs for passive smoking not vary significantly by location ^73^, but even if passive smoking exposure were particularly common in Japanese nonsmokers, the relatively weak association of passive smoking with lung cancer risk could not possibly explain why active smoking relative risks are two-fold or more higher in Western than in Asian populations.

## Conclusions

In Japanese studies, smoking is related to an increased risk of all four diseases studied, though the increase is relatively weak for stroke, and the evidence is limited for COPD. For IHD, the estimated RR for current smoking, of 2.21 (95%CI 1.96–2.50) is similar to that reported in other Asian and in Western populations and is dose-related, increasing with amount smoked and reducing with years quit. For lung cancer, the estimated RR for current smoking of 3.59 (3.25–3.96), which is also clearly dose-related, is similar to that in other Asian populations but substantially less than in Western populations. The explanation of this difference is unclear but high rates of denial of cigarette smoking may contribute.

## Data availability

All data underlying the results are available as part of the article and no additional source data are required.

## Supplementary material

**Supplementary File 1: Completed PRISMA checklist.**

**Supplementary File 2: Searches conducted.** This document gives more details on the searches conducted.

**Supplementary File 3: Relative risks by amount smoked and duration of quitting.**

**Supplementary File 4: More detailed meta-analysis results for cardiovascular disease and lung cancer.**
