# Supplementary material for: The relationship of cigarette smoking in Japan to lung cancer, COPD, ischemic heart disease and stroke: A systematic review
Source: F1000Res. 2018 Feb 19;7:204. [Version 1] doi: 10.12688/f1000research.14002.1 (PMC6367657; doi:10.12688/f1000research.14002.1)
Supplement: Supplementary file 2 [file f1000research-7-15218-s0001.tgz › 2d1ad835-afe6-4bfb-bf02-379372a2d221.docx]

**Supplementary File 1**

**Searches conducted**

This document gives more details on the searches conducted. Selected studies are referred to by the study reference code, as shown in Table 1 of the main paper, which also gives the associated source references.

Lung cancer

A review published in 2012^1^ of the evidence relating smoking to lung cancer published in the 1990s cited 19 studies in Japan. Eleven of these (AKIBA, GAO2, HIRAYA, HITOSU, KIHARA, SEGI2, SOBUE, SOBUE2, TSUGAN, WAKAI, YAMAGU) are included in our analyses. Eight were not. Four did not present results for current smoking (original study IDs ESAKI, ISHIMA, MATSUD, SEGI) three did not present RRs relative to never smokers (HIRAY2, MURATA, SHIMIZ) and one only presented results for a specific lung cancer type (SUZUKI). Three additional relevant studies (OSAKI, STELL1, WAKAI2) were identified from a recent review of the decline in risk of lung cancer following quitting^2^.

A further Medline search on smoking and lung cancer restricted to Japan and humans and to publications from 2000 onwards produced 836, of which 760 were rejected based on the abstract; and two were already identified from the quitting review. Of the remaining 74 papers, 29 were rejected for various reasons: no results for never smokers^3^, no relevant data^4-19^, data for adenocarcinoma only^20 21^, cross-sectional study^22^, <100 cases^23-25^, pooled analysis with no separate results by study^26^, study of cancer survival^27 28^ and review paper not of interest^29-31^. There was also one review of interest^32^ but no additional useful references were found. The remaining 44 papers related to only 13 studies. The study references, main reference, and additional references providing no useful extra data are shown below.

| **Study** | **Main reference** | **Additional references** |
| --- | --- | --- |
| AKIBA* | ^33^* | ^34^ |
| FUKUMO | ^35^ | ^36^ |
| HIRAKI | ^37^ | ^38^ |
| ITO | ^39^ | ^40-47^ |
| KANASH | ^48^ | - |
| KAWAMI | ^49^ | - |
| KIYOHA | ^50^ | ^51-54^ |
| MARUG1 | ^55^ | - |
| MARUG2 | ^56^ | - |
| MINAMI | ^57^ | ^58^ |
| OZASA | ^59^ | ^60-69^ |
| SHIMIZ** | ^70^ | ^71-75^ |
| SOBUE3 | ^76^ | - |

* Note that EGAWA2012 provides data for the earlier AKIBA study.

** Note that the five additional references are also relevant to SOBUE3.

COPD

A review published in 2011^77^ of the evidence relating smoking to COPD, chronic bronchitis and emphysema included four relevant studies in Japan on COPD. Two (FUKUCH, TSUSHI) were included in our analyses, but two were rejected, one^78^ as it had no relevant data on current or ex-smoking and one^79^ as it was superseded by a more recent publication found in our updated searches. No additional relevant studies in Japan were identified from a recent review of the decline in risk of COPD following quitting^80^.

Further Medline searches were conducted using the same terms as used in the 2011 review^77^, but restricted to Japan and to the period from 2007 onwards. This yielded 105 hits, of which 83 were rejected based on the abstract. Of the 22 papers examined, 17 were rejected for various reasons, leaving five extra studies (HIRAY2, HORIE, KOJIM2, OMORI2, OSAKA). Reasons for rejection included the following: No data on current or former smoking^81-83^, no never smokers^84 85^, not COPD as defined^86-89^, patients with rotator cuff tears^90^, results already reported in an earlier paper^91^, no smoking data for those without COPD^92^, no non-ETS exposed nonsmokers^93^, no relevant data^94 95^, risks only expressed as pack-years^96^ and only a review paper citing no new references^97^.

Heart disease and stroke

A recent publication^98^ includes as a Supplementary File details of a review conducted in 2010 based on epidemiological studies conducted in Europe, Asia or North America involving at least 100 cases of cardiovascular disease, limited to papers published since 1990. This review included four publications relating to studies in Japan, three describing results from individual studies (HIRAYA, YAMAGI, UESHIM) and one of which was a pooled analysis (3 STUDIES). No additional relevant studies in Japan were identified from a recent review of the decline in risk following quitting for heart disease^99^ while one was identified from the equivalent review on stroke^100^ (KONDO).

Further Medline searches were conducted to look for additional studies, by linking “smoking” and “Japan” to the various disease terms used in our earlier reviews^99 100^. This yielded 618 hits, 524 being rejected based on the abstract. Of the 94 papers examined, one was the pooled analysis referred to above, one was the paper identified from the stroke review and 61 were rejected on initial examination, many as smoking was only considered as an adjustment factor, with no smoking results being presented. The remaining 31 were then examined to check that they fully satisfied the inclusion criteria of our recently published review^98^. Nineteen of the 31 new papers failed the additional criteria, one only reporting results for ischaemic stroke^101^, one not reporting results by smoking^102^, three not providing adjusted results^103-105^, 12 not reporting results by sex^106-117^ and two not reporting adjusted results or results by sex^118 119^. The original five papers (HIRAYA, YAMAGI, UESHIM, 3 STUDIES, KONDO) and the other 12 new papers were then examined to check the diseases they presented results for, and the extent to which they concerned the same or different studies. Of the 12 new papers, three^120-122^ were rejected as they only provided data for combined cardiovascular disease and four^123-126^ were rejected as they did not provide risk estimates relative to never smokers. One further paper^127^ was rejected as a later paper^128^ provided better results from the JACC study. Three of the remaining four new papers provided relevant material for individual studies (JPHC, HATANA, JACC) while one presented a pooled analysis (10 STUDIES).

References

1. Lee PN, Forey BA, Coombs KJ. Systematic review with meta-analysis of the epidemiological evidence in the 1900s relating smoking to lung cancer. *BMC Cancer* 2012;**12**:385.

2. Fry JS, Lee PN, Forey BA, Coombs KJ. How rapidly does the excess risk of lung cancer decline following quitting smoking? A quantitative review using the negative exponential model. *Regul Toxicol Pharmacol* 2013;**67**:13-26.

3. Ariyoshi N, Miyamoto M, Umetsu Y, Kunitoh H, Dosaka-Akita H, Sawamura Y-i, et al. Genetic polymorphism of *CYP2A6* gene and tobacco-induced lung cancer risk in male smokers. *Cancer Epidemiol Biomarkers Prev* 2002;**11**(9):890-4.

4. Hamajima N, Matsuo K, Iwata H, Shinoda M, Yamamura Y, Kato T, et al. NAD(P)H: quinone oxidoreductase 1 (NQO1) C609T polymorphism and the risk of eight cancers for Japanese. *Int J Clin Oncol* 2002;**7**(2):103-8.

5. Ito H, Matsuo K, Tanaka H, Koestler DC, Ombao H, Fulton J, et al. Nonfilter and filter cigarette consumption and the incidence of lung cancer by histological type in Japan and the United States: analysis of 30-year data from population-based cancer registries. *Int J Cancer* 2011;**128**(8):1918-28.

6. Katanoda K, Sobue T, Satoh H, Tajima K, Suzuki T, Nakatsuka H, et al. An association between long-term exposure to ambient air pollution and mortality from lung cancer and respiratory diseases in Japan. *J Epidemiol* 2011;**21**(2):132-43.

7. Kawaguchi T, Ando M, Ito N, Isa S, Tamiya A, Shimizu S, et al. Rationale and design of the Japan molecular epidemiology for lung cancer study. *Clin Lung Cancer* 2013;**14**(5):596-600.

8. Sawada N, Iwasaki M, Inoue M, Takachi R, Sasazuki S, Yamaji T, et al. Dietary arsenic intake and subsequent risk of cancer: the Japan Public Health Center-based (JPHC) Prospective Study. *Cancer Causes Control* 2013;**24**(7):1403-15.

9. Tsuda T, Mino Y, Babazono A, Shigemi J, Otsu T, Yamamoto E, et al. A case-control study of lung cancer in relation to silica exposure and silicosis in a rural area in Japan. *Ann Epidemiol* 2002;**12**(5):288-94.

10. Uehara Y, Kiyohara C. Alcohol consumption and lung cancer risk among Japanese: a meta-analysis. *Fukuoka Igaku Zasshi* 2010;**101**(5):101-8.

11. Nagano J, Kono S, Preston DL, Mabuchi K. A prospective study of green tea consumption and cancer incidence, Hiroshima and Nagasaki (Japan). *Cancer Causes Control* 2001;**12**:501-8.

12. Wakai K, Sugawara Y, Tsuji I, Tamakoshi A, Shimazu T, Matsuo K, et al. Risk of lung cancer and consumption of vegetables and fruit in Japanese: A pooled analysis of cohort studies in Japan. *Cancer Sci* 2015;**106**(8):1057-65.

13. Huang X-E, Hirose K, Wakai K, Matsuo K, Ito H, Xiang J, et al. Comparison of lifestyle risk factors by family history for gastric, breast, lung and colorectal cancer. *Asian Pac J Cancer Prev* 2004;**5**(4):419-27.

14. Kanzaki H, Ouchida M, Hanafusa H, Yano M, Suzuki H, Aoe M, et al. Single nucleotide polymorphism of the AXIN2 gene is preferentially associated with human lung cancer risk in a Japanese population. *Int J Mol Med* 2006;**18**(2):279-84.

15. Miyaishi A, Osawa K, Osawa Y, Inoue N, Yoshida K, Kasahara M, et al. *MUTYH* Gln324His gene polymorphism and genetic susceptibility for lung cancer in a Japanese population. *J Exp Clin Cancer Res* 2009;**28**:10.

16. Osawa Y, Osawa K, Miyaishi A, Higuchi M, Tsutou A, Matsumura S, et al. NAT2 and CYP1A2 polymorphisms and lung cancer risk in relation to smoking status. *Asian Pac J Cancer Prev* 2007;**8**(1):103-8.

17. Osawa K, Miyaishi A, Uchino K, Osawa Y, Inoue N, Nakarai C, et al. APEX1 Asp148Glu gene polymorphism is a risk factor for lung cancer in relation to smoking in Japanese. *Asian Pac J Cancer Prev* 2010;**11**(5):1181-6.

18. Shiraishi K, Kohno T, Kunitoh H, Watanabe S, Goto K, Nishiwaki Y, et al. Contribution of nicotine acetylcholine receptor polymorphisms to lung cancer risk in a smoking-independent manner in the Japanese. *Carcinogenesis* 2009;**30**(1):65-70.

19. Tamaki Y, Arai T, Sugimura H, Sasaki T, Honda M, Muroi Y, et al. Association between cancer risk and drug-metabolizing enzyme gene (*CYP2A6, CYP2A13, CYP4B1, SULT1A1, GSTM1,* and *GSTT1*) polymorphisms in cases of lung cancer in Japan. *Drug Metab Pharmacokinet* 2011;**26**(5):516-22.

20. Ito H, Hamajima N, Takezaki T, Matsuo K, Tajima K, Hatooka S, et al. A limited association of OGG1 Ser326Cys polymorphism for adenocarcinoma of the lung. *J Epidemiol* 2002;**12**(3):258-65.

21. Sunaga N, Kohno T, Yanagitani N, Sugimura H, Kunitoh H, Tamura T, et al. Contribution of the *NQ01* and *GSTT1* polymorphisms to lung adenocarcinoma susceptibility. *Cancer Epidemiol Biomarkers Prev* 2002;**11**:730-8.

22. Henschke CI, Yip R, Boffetta P, Markowitz S, Miller A, Hanaoka T, et al. CT screening for lung cancer: Importance of emphysema for never smokers and smokers. *Lung Cancer* 2015;**88**(1):42-7.

23. Kishi K, Gurney JW, Schroeder DR, Scanlon PD, Swensen SJ, Jett JR. The correlation of emphysema or airway obstruction with the risk of lung cancer: a matched case-controlled study. *Eur Respir J* 2002;**19**(6):1093-8.

24. Nojo T, Imanaka Y, Ishizaki T, Sekimoto M, Yoshino M, Kurosawa T, et al. Lung cancer incidence in middle-aged men estimated by low-dose computed tomography screening. *Lung Cancer* 2009;**65**(1):56-61.

25. Courtice MN, Wang X, Lin S, Yu IT, Berman DW, Yano E. Exposure-response estimate for lung cancer and asbestosis in a predominantly chrysotile-exposed Chinese factory cohort. *Am J Ind Med* 2016;**59**(5):369-78. Available from: <http://onlinelibrary.wiley.com/doi/10.1002/ajim.22579/abstract>.

26. Zheng W, McLerran DF, Rolland BA, Fu Z, Boffetta P, He J, et al. Burden of total and cause-specific mortality related to tobacco smoking among adults aged >/= 45 years in Asia: a pooled analysis of 21 cohorts. *PLoS Med* 2014;**11**(4):e1001631.

27. Tabuchi T, Ito Y, Ioka A, Nakayama T, Miyashiro I, Tsukuma H. Tobacco smoking and the risk of subsequent primary cancer among cancer survivors: a retrospective cohort study. *Ann Oncol* 2013;**24**(10):2699-704.

28. Tabuchi T, Ozaki K, Ioka A, Miyashiro I. Joint and independent effect of alcohol and tobacco use on the risk of subsequent cancer incidence among cancer survivors: A cohort study using cancer registries. *Int J Cancer* 2015;**137**(9):2114-23.

29. Takahashi I, Matsuzaka M, Umeda T, Yamai K, Nishimura M, Danjo K, et al. Differences in the influence of tobacco smoking on lung cancer between Japan and the USA: possible explanations for the 'smoking paradox' in Japan. *Public Health* 2008;**122**(9):891-6.

30. Tanaka H. Advances in cancer epidemiology in Japan. *Int J Cancer* 2014;**134**(4):747-54.

31. Wakai K, Nagata C, Mizoue T, Tanaka K, Nishino Y, Tsuji I, et al. Alcohol drinking and lung cancer risk: an evaluation based on a systematic review of epidemiologic evidence among the Japanese population. *Jpn J Clin Oncol* 2007;**37**(3):168-74.

32. Wakai K, Inoue M, Mizoue T, Tanaka K, Tsuji I, Nagata C, et al. Tobacco smoking and lung cancer risk: an evaluation based on a systematic review of epidemiological evidence among the Japanese population. *Jpn J Clin Oncol* 2006;**36**(5):309-24.

33. Egawa H, Furukawa K, Preston D, Funamoto S, Yonehara S, Matsuo T, et al. Radiation and smoking effects on lung cancer incidence by histological types among atomic bomb survivors. *Radiat Res* 2012;**178**(3):191-201.

34. Furukawa K, Preston DL, Lönn S, Funamoto S, Yonehara S, Matsuo T, et al. Radiation and smoking effects on lung cancer incidence among atomic bomb survivors. *Radiat Res* 2010;**174**(1):72-82.

35. Fukumoto K, Ito H, Matsuo K, Tanaka H, Yokoi K, Tajima K, et al. Cigarette smoke inhalation and risk of lung cancer: a case-control study in a large Japanese population. *Eur J Cancer Prev* 2015;**24**(3):195-200.

36. Muscat JE, Takezaki T, Tajima K, Stellman ST. Charcoal cigarette filters and lung cancer risk in Aichi Prefecture, Japan. *Cancer Sci* 2005;**96**(5):283-7.

37. Hiraki A, Matsuo K, Hamajima N, Ito H, Hatooka S, Suyama M, et al. Different risk relations with smoking for non-small-cell lung cancer: comparison of TP53 and TP73 genotypes. *Asian Pac J Cancer Prev* 2003;**4**(2):107-12.

38. Kumimoto H, Hamajima N, Nishimoto Y, Matsuo K, Shinoda M, Hatooka S, et al. *L-myc* genotype is associated with different susceptibility to lung cancer in smokers. *Jpn J Cancer Res* 2002;**93**(1):1-5.

39. Ito H, Gallus S, Hosono S, Oze I, Fukumoto K, Yatabe Y, et al. Time to first cigarette and lung cancer risk in Japan. *Ann Oncol* 2013;**24**(11):2870-5.

40. Ebi H, Oze I, Nakagawa T, Ito H, Hosono S, Matsuda F, et al. Lack of association between the BIM deletion polymorphism and the risk of lung cancer with and without EGFR mutations. *J Thorac Oncol* 2015;**10**(1):59-66.

41. Ito H, Matsuo K, Hamajima N, Mitsudomi T, Sugiura T, Saito T, et al. Gene-environment interactions between the smoking habit and polymorphisms in the DNA repair genes, APE1 Asp148Glu and XRCC1 Arg399Gln, in Japanese lung cancer risk. *Carcinogenesis* 2004;**25**(8):1395-401.

42. Ito H, McKay JD, Hosono S, Hida T, Yatabe Y, Mitsudomi T, et al. Association between a genome-wide association study-identified locus and the risk of lung cancer in Japanese population. *J Thorac Oncol* 2012;**7**(5):790-8.

43. Matsuo K, Ito H, Yatabe Y, Hiraki A, Hirose K, Wakai K, et al. Risk factors differ for non-small-cell lung cancers with and without EGFR mutation: assessment of smoking and sex by a case-control study in Japanese. *Cancer Sci* 2007;**98**(1):96-101.

44. Nakane H, Hirano M, Ito H, Hosono S, Oze I, Matsuda F, et al. Impact of metallothionein gene polymorphisms on the risk of lung cancer in a Japanese population. *Mol Carcinog* 2015;**54(Suppl 1)**:E122-8. Erratum appears in Mol Carcinog, 2015, 54, 935.

45. Okasaka T, Matsuo K, Suzuki T, Ito H, Hosono S, Kawase T, et al. hOGG1 Ser326Cys polymorphism and risk of lung cancer by histological type. *J Hum Genet* 2009;**54**(12):739-45.

46. Park JY, Matsuo K, Suzuki T, Ito H, Hosono S, Kawase T, et al. Impact of smoking on lung cancer risk is stronger in those with the homozygous aldehyde dehydrogenase 2 null allele in a Japanese population. *Carcinogenesis* 2010;**31**(4):660-5.

47. Suzuki T, Matsuo K, Hiraki A, Saito T, Sato S, Yatabe Y, et al. Impact of one-carbon metabolism-related gene polymorphisms on risk of lung cancer in Japan: a case control study. *Carcinogenesis* 2007;**28**(8):1718-25.

48. Kanashiki M, Sairenchi T, Saito Y, Ishikawa H, Ishikawa H, Satoh H, et al. Body mass index and lung cancer: a case-control study of subjects participating in a mass-screening program. *Chest* 2005;**128**(3):1490-6.

49. Kawaminami K, Minowa M, Okayama A, Hayakawa T. (An association [population attributable fraction] between smoking habit and mortality from all causes, cancer and lung cancer: NIPPON DATA80, 1980-1999. National integrated projects for prospective observation of non-communicable diseases and its trend in the aged). *Nihon Eiseigaku Zasshi* 2003;**57**(4):669-73.

50. Kiyohara C, Horiuchi T, Takayama K, Nakanishi Y. IL1B rs1143634 polymorphism, cigarette smoking, alcohol use, and lung cancer risk in a Japanese population. *J Thorac Oncol* 2010;**5**(3):299-304.

51. Kakino K, Kiyohara C, Horiuchi T, Nakanishi Y. CYP2E1 rs2031920, COMT rs4680 polymorphisms, cigarette smoking, alcohol use and lung cancer risk in a Japanese population. *Asian Pac J Cancer Prev* 2016;**17**(8):4063-70.

52. Kiyohara C, Horiuchi T, Miyake Y, Takayama K, Nakanishi Y. Cigarette smoking, TP53 Arg72Pro, TP53BP1 Asp353Glu and the risk of lung cancer in a Japanese population. *Oncol Rep* 2010;**23**(5):1361-8.

53. Kiyohara C, Horiuchi T, Takayama K, Nakanishi Y. Methylenetetrahydrofolate reductase polymorphisms and interaction with smoking and alcohol consumption in lung cancer risk: a case-control study in a Japanese population. *BMC Cancer* 2011;**11**:459.

54. Kiyohara C, Horiuchi T, Takayama K, Nakanishi Y. Genetic polymorphisms involved in the inflammatory response and lung cancer risk: a case-control study in Japan. *Cytokine* 2014;**65**(1):88-94.

55. Marugame T, Sobue T, Satoh H, Komatsu S, Nishino Y, Nakatsuka H, et al. Lung cancer death rates by smoking status: comparison of the Three-Prefecture Cohort study in Japan to the Cancer Prevention Study II in the USA. *Cancer Sci* 2005;**96**:120-6.

56. Marugame T, Sobue T, Nakayama T, Suzuki T, Kuniyoshi H, Sunagawa K, et al. Filter cigarette smoking and lung cancer risk; a hospital-based case-control study in Japan. *Br J Cancer* 2004;**90**:646-51.

57. Minami Y, Tateno H. Associations between cigarette smoking and the risk of four leading cancers in Miyagi Prefecture, Japan: a multi-site case-control study. *Cancer Sci* 2003;**94**(6):540-7.

58. Seki T, Nishino Y, Tanji F, Maemondo M, Takahashi S, Sato I, et al. Cigarette smoking and lung cancer risk according to histologic type in Japanese men and women. *Cancer Sci* 2013;**104**(11):1515-22.

59. Ozasa K. Smoking and mortality in the Japan Collaborative Cohort Study for Evaluation of Cancer (JACC). *Asian Pac J Cancer Prev* 2007;**8(Suppl)**:89-96.

60. Ando M, Wakai K, Seki N, Tamakoshi A, Suzuki K, Ito Y, et al. Attributable and absolute risk of lung cancer death by smoking status: findings from the Japan Collaborative Cohort Study. *Int J Cancer* 2003;**105**(2):249-54.

61. Ito Y, Wakai K, Suzuki K, Tamakoshi A, Seki N, Ando M, et al. Serum carotenoids and mortality from lung cancer: a case-control study nested in the Japan Collaborative Cohort (JACC) study. *Cancer Sci* 2003;**94**(1):57-63.

62. Ito Y, Wakai K, Suzuki K, Ozasa K, Watanabe Y, Seki N, et al. Lung cancer mortality and serum levels of carotenoids, retinol, tocopherols, and folic acid in men and women: a case-control study nested in the JACC Study. *J Epidemiol* 2005;**15(Suppl 2)**:S140-S9.

63. Kondo T, Hori Y, Yatsuya H, Tamakoshi K, Toyoshima H, Nishino Y, et al. Lung cancer mortality and body mass index in a Japanese cohort: findings from the Japan Collaborative Cohort Study (JACC Study). *Cancer Causes Control* 2007;**18**(2):229-34.

64. Nishino Y, Wakai K, Kondo T, Seki N, Ito Y, Suzuki K, et al. Alcohol consumption and lung cancer mortality in Japanese men: results from Japan Collaborative Cohort (JACC) study. *J Epidemiol* 2006;**16**:49-56.

65. Ozasa K, Watanabe Y, Ito Y, Suzuki K, Tamakoshi A, Seki N, et al. Dietary habits and risk of lung cancer death in a large-scale cohort study (JACC Study) in Japan by sex and smoking habit. *Jpn J Cancer Res* 2001;**92**(12):1259-69.

66. Suzuki K, Ito Y, Wakai K, Kawado M, Hashimoto S, Seki N, et al. Serum heat shock protein 70 levels and lung cancer risk: a case-control study nested in a large cohort study. *Cancer Epidemiol Biomarkers Prev* 2006;**15**(9):1733-7.

67. Wakai K, Seki N, Tamakoshi A, Kondo T, Nishino Y, Ito Y, et al. Decrease in risk of lung cancer death in males after smoking cessation by age at quitting: findings from the JACC study. *Jpn J Cancer Res* 2001;**92**(8):821-8.

68. Wakai K, Ito Y, Suzuki K, Tamakoshi A, Seki N, Ando M, et al. Serum insulin-like growth factors, insulin-like growth factor-binding protein-3, and risk of lung cancer death: a case-control study nested in the Japan Collaborative Cohort (JACC) Study. *Jpn J Cancer Res* 2002;**93**(12):1279-86.

69. Wakai K, Ando M, Ozasa K, Ito Y, Suzuki K, Nishino Y, et al. Updated information on risk factors for lung cancer: findings from the JACC study. *J Epidemiol* 2005;**15(Suppl II)**:S134-S9.

70. Shimazu T, Inoue M, Sasazuki S, Iwasaki M, Sawada N, Yamaji T, et al. Isoflavone intake and risk of lung cancer: a prospective cohort study in Japan. *Am J Clin Nutr* 2010;**91**(3):722-8.

71. Hara M, Inoue M, Shimazu T, Yamamoto S, Tsugane S. The association between cancer risk and age at onset of smoking in Japanese. *J Epidemiol* 2010;**20**(2):128-35.

72. Liu Y, Sobue T, Otani T, Tsugane S. Vegetables, fruit consumption and risk of lung cancer among middle-aged Japanese men and women: JPHC study. *Cancer Causes Control* 2004;**15**(4):349-57.

73. Nitadori J, Inoue M, Iwasaki M, Otani T, Sasazuki S, Nagai K, et al. Association between lung cancer incidence and family history of lung cancer: data from a large-scale population-based cohort, the JPHC study. *Chest* 2006;**130**:968-75.

74. Shimazu T, Inoue M, Sasazuki S, Iwasaki M, Kurahashi N, Yamaji T, et al. Alcohol and risk of lung cancer among Japanese men: data from a large-scale population-based cohort study, the JPHC study. *Cancer Causes Control* 2008;**19**(10):1095-102.

75. Shimazu T, Inoue M, Sasazuki S, Iwasaki M, Sawada N, Yamaji T, et al. Plasma isoflavones and the risk of lung cancer in women: a nested case-control study in Japan. *Cancer Epidemiol Biomarkers Prev* 2011;**20**(3):419-27.

76. Sobue T, Yamamoto S, Hara M, Sasazuki S, Sasaki S, Tsugane S. Cigarette smoking and subsequent risk of lung cancer by histologic type in middle-aged Japanese men and women: the JPHC study. *Int J Cancer* 2002;**99**(2):245-51.

77. Forey BA, Thornton AJ, Lee PN. Systematic review with meta-analysis of the epidemiological evidence relating smoking to COPD, chronic bronchitis and emphysema. *BMC Pulm Med* 2011;**11**:36.

78. Itabashi S, Fukushima T, Aikawa T, Yanai M, Sekizawa K, Sasaki H, et al. Allergic sensitization in elderly patients with chronic obstructive pulmonary disease. *Respiration* 1990;**57**:384-8.

79. Kojima S, Sakakibara H, Motani S, Hirose K, Mizuno F, Ito M, et al. Effects of smoking and age on chronic obstructive pulmonary disease in Japan. *J Epidemiol* 2005;**15**(4):113-7.

80. Lee PN, Fry JS, Forey BA. Estimating the decline in excess risk of chronic obstructive pulmonary disease following quitting smoking - a systematic review based on the negative exponential model. *Regul Toxicol Pharmacol* 2014;**68**(2):231-9.

81. Hirayama F, Lee AH, Terasawa K, Kagawa Y. Folate intake associated with lung function, breathlessness and the prevalence of chronic obstructive pulmonary disease. *Asia Pac J Clin Nutr* 2010;**19**(1):103-9.

82. Matsumoto K, Seki N, Fukuyama S, Moriwaki A, Kan-o K, Matsunaga Y, et al. Prevalence of asthma with airflow limitation, COPD, and COPD with variable airflow limitation in older subjects in a general Japanese population: the Hisayama Study. *Respir. Investig.* 2015;**53**(1):22-9.

83. Yamane T, Hattori N, Kitahara Y, Haruta Y, Sasaki H, Yokoyama A, et al. Productive cough is an independent risk factor for the development of COPD in former smokers. *Respirology* 2010;**15**(2):313-8.

84. Higashimoto Y, Yamagata Y, Taya S, Iwata T, Okada M, Ishiguchi T, et al. Systemic inflammation in chronic obstructive pulmonary disease and asthma: Similarities and differences. *Respirology* 2008;**13**(1):128-33.

85. Ishii T, Wakabayashi R, Kurosaki H, Gemma A, Kida K. Association of serotonin transporter gene variation with smoking, chronic obstructive pulmonary disease, and its depressive symptoms. *J Hum Genet* 2011;**56**(1):41-6.

86. Akamatsu K, Yamagata T, Kida Y, Tanaka H, Ueda H, Ichinose M. Poor sensitivity of symptoms in early detection of COPD. *COPD* 2008;**5**(5):269-73.

87. Bando M, Miyazawa T, Shinohara H, Owada T, Terakado M, Sugiyama Y. An epidemiological study of the effects of statin use on airflow limitation in patients with chronic obstructive pulmonary disease. *Respirology* 2012;**17**(3):493-8.

88. Togami T, Yamamoto Y, Satoh K, Kameyama R, Murota M, Kimura N, et al. CT assessment of subtypes of pulmonary emphysema in females. *Acta Med Okayama* 2011;**65**(1):27-32.

89. Horiuchi N, Fujita J, Suemitsu I, Yamasaki Y, Higa F, Tateyama M. Low-dose multislice CT and high-resolution CT assessment of pulmonary emphysema in public school teachers. *Lung* 2007;**185**(1):25-30.

90. Yoshii C, Uchida S, Noguchi S, Torii R, Shimabukuro I, Yatera K. Smoking Status and a Pulmonary Function Test in Patients with Rotator Cuff Tears. *J UOEH* 2016;**38**(3):243-9.

91. Tsushima K, Sone S, Fujimoto K, Kubo K, Morita S, Takegami M, et al. Identification of occult parechymal disease such as emphysema or airway disease using screening computed tomography. *COPD* 2010;**7**(2):117-25.

92. Wada H, Nakano Y, Nagao T, Osawa M, Yamada H, Sakaguchi C, et al. Detection and prevalence of chronic obstructive pulmonary disease in a cardiovascular clinic: evaluation using a hand held FEV(1)/FEV(6) meter and questionnaire. *Respirology* 2010;**15**(8):1252-8.

93. Sekine Y, Yanagibori R, Suzuki K, Sugiyama S, Yamaji H, Ishibashi M, et al. Surveillance of chronic obstructive pulmonary disease in high-risk individuals by using regional lung cancer mass screening. *Int J Chron Obstruct Pulmon Dis* 2014;**9**:647-56.

94. Galvan A, Dragani TA. Nicotine dependence may link the 15q25 locus to lung cancer risk. *Carcinogenesis* 2010;**31**(3):331-3.

95. Yoshimoto D, Nakano Y, Onishi K, Hagan G, Jones P. The relationship between the COPD Assessment Test score and airflow limitation in Japan in patients aged over 40 years with a smoking history. *Int J Chron Obstruct Pulmon Dis* 2014;**9**:1357-63.

96. Hirayama F, Lee AH. Association between childhood asthma and chronic obstructive pulmonary disease in later life. *Asia Pac J Public Health* 2015;**27**(2):Np1273-9.

97. Rycroft CE, Heyes A, Lanza L, Becker K. Epidemiology of chronic obstructive pulmonary disease: a literature review. *Int J Chron Obstruct Pulmon Dis* 2012;**7**:457-94.

98. Lee PN, Fry JS, Hamling JF, Sponsiello-Wang Z, Baker G, Weitkunat R. Estimating the effect of differing assumptions on the population health impact of introducing a Reduced Risk Tobacco Product in the USA. *Regul Toxicol Pharmacol* 2017;**88**:192-213.

99. Lee PN, Fry JS, Hamling JS. Using the negative exponential distribution to quantitatively review the evidence on how rapidly the excess risk of ischaemic heart disease declines following quitting smoking. *Regul Toxicol Pharmacol* 2012;**64**:51-67.

100. Lee PN, Fry JS, Thornton A. Estimating the decline in excess risk of cerebrovascular disease following quitting smoking - A systematic review based on the negative exponential model. *Regul Toxicol Pharmacol* 2014;**68**(1):85-95.

101. Yonemoto K, Doi Y, Hata J, Ninomiya T, Fukuhara M, Ikeda F, et al. Body mass index and stroke incidence in a Japanese community: the Hisayama study. *Hypertens Res* 2011;**34**(2):274-9.

102. Hashimoto T, Kikuya M, Ohkubo T, Satoh M, Metoki H, Inoue R, et al. Home blood pressure level, blood pressure variability, smoking, and stroke risk in Japanese men: the Ohasama study. *Am J Hypertens* 2012;**25**(8):883-91.

103. Manabe Y, Morihara R, Matsuzono K, Nakano Y, Takahashi Y, Narai H, et al. Estimation of the presence of small dense lipoprotein cholesterol in acute ischemic stroke. *Neurol Int* 2015;**7**(1):5973.

104. Saito I, Iso H, Kokubo Y, Inoue M, Tsugane S. Body mass index, weight change and risk of stroke and stroke subtypes: the Japan Public Health Center-based prospective (JPHC) study. *Int J Obes (Lond)* 2010;**35**(2):283-91.

105. Yatsuya H, Iso H, Yamagishi K, Kokubo Y, Saito I, Suzuki K, et al. Development of a point-based prediction model for the incidence of total stroke: Japan public health center study. *Stroke* 2013;**44**(5):1295-302.

106. Teramoto T, Kawamori R, Miyazaki S, Teramukai S, Mori Y, Okuda Y, et al. Risk factors for primary prevention of cardiovascular disease and risk reduction by lipid control: the OMEGA study risk factor sub-analysis. *Clin Exp Hypertens* 2014;**36**(4):236-43.

107. Hata J, Doi Y, Ninomiya T, Fukuhara M, Ikeda F, Mukai N, et al. Combined effects of smoking and hypercholesterolemia on the risk of stroke and coronary heart disease in Japanese: the Hisayama study. *Cerebrovasc Dis* 2011;**31**(5):477-84.

108. Hokari M, Isobe M, Imai T, Chiba Y, Iwamoto N, Isu T. The impact of atherosclerotic factors on cerebral aneurysm is location dependent: aneurysms in stroke patients and healthy controls. *J Stroke Cerebrovasc Dis* 2014;**23**(9):2301-7.

109. Kitamura A, Yamagishi K, Imano H, Kiyama M, Cui R, Ohira T, et al. Impact of hypertension and subclinical organ damage on the incidence of cardiovascular disease among Japanese residents at the population and individual levels- The Circulatory Risk in Communities Study (CIRCS). *Circ J* 2017;**81**(7):1022-8.

110. Kondo H, Ninomiya T, Hata J, Hirakawa Y, Yonemoto K, Arima H, et al. Angiotensin I-converting enzyme gene polymorphism enhances the effect of hypercholesterolemia on the risk of coronary heart disease in a general Japanese population: the hisayama study. *J Atheroscler Thromb* 2015;**22**(4):390-403.

111. Miyazaki T, Shimada K, Hiki M, Kume A, Kitamura Y, Oshida K, et al. High hexacosanoic acid levels are associated with coronary artery disease. *Atherosclerosis* 2014;**233**(2):429-33.

112. Morita K, Miyazaki H, Saruwatari J, Oniki K, Kumagae N, Tanaka T, et al. Combined effects of current-smoking and the aldehyde dehydrogenase 2*2 allele on the risk of myocardial infarction in Japanese patients. *Toxicol Lett* 2015;**232**(1):221-5.

113. Ohira T, Maruyama M, Imano H, Kitamura A, Kiyama M, Okada T, et al. Risk factors for sudden cardiac death among Japanese: the Circulatory Risk in Communities Study. *J Hypertens* 2012;**30**(6):1137-43.

114. Palatini P, Reboldi G, Beilin LJ, Eguchi K, Imai Y, Kario K, et al. Predictive value of night-time heart rate for cardiovascular events in hypertension. The ABP-International study. *Int J Cardiol* 2013;**168**(2):1490-5.

115. Senmaru T, Fukui M, Tanaka M, Kuroda M, Yamazaki M, Oda Y, et al. Atrophic gastritis is associated with coronary artery disease. *J Clin Biochem Nutr* 2012;**51**(1):39-41.

116. Yamase Y, Horibe H, Ueyama C, Fujimaki T, Oguri M, Kato K, et al. Association of TOMM40 and SLC22A4 polymorphisms with ischemic stroke. *Biomed Rep* 2015;**3**(4):491-8.

117. Yatsuya H, Iso H, Li Y, Yamagishi K, Kokubo Y, Saito I, et al. Development of a risk equation for the incidence of coronary artery disease and ischemic stroke for middle-aged Japanese- Japan Public Health Center-Based Prospective Study. *Circ J* 2016;**80**(6):1386-95.

118. Ueno M, Izumi Y, Kawaguchi Y, Ikeda A, Iso H, Inoue M, et al. Prediagnostic plasma antibody levels to periodontopathic bacteria and risk of coronary heart disease. *Int Heart J* 2012;**53**(4):209-14.

119. Yamada Y, Matsui K, Takeuchi I, Fujimaki T. Association of genetic variants with coronary artery disease and ischemic stroke in a longitudinal population-based genetic epidemiological study. *Biomed Rep* 2015;**3**(3):413-9.

120. Kondo N, Saito M, Hikichi H, Aida J, Ojima T, Kondo K, et al. Relative deprivation in income and mortality by leading causes among older Japanese men and women: AGES cohort study. *J Epidemiol Community Health* 2015;**69**(7):680-5.

121. Kokubo Y, Iso H, Saito I, Yamagishi K, Ishihara J, Inoue M, et al. Dietary fiber intake and risk of cardiovascular disease in the Japanese population: the Japan Public Health Center-based study cohort. *Eur J Clin Nutr* 2011;**65**(11):1233-41.

122. Eguchi E, Iso H, Tanabe N, Wada Y, Yatsuya H, Kikuchi S, et al. Healthy lifestyle behaviours and cardiovascular mortality among Japanese men and women: the Japan collaborative cohort study. *Eur Heart J* 2012;**33**(4):467-77.

123. Iwahana H, Ishikawa S, Ishikawa J, Kabutoya T, Kayaba K, Gotoh T, et al. Atrial fibrillation is a major risk factor for stroke, especially in women: the Jichi Medical School cohort study. *J Epidemiol* 2011;**21**(2):95-101.

124. Morimoto T, Mineharu Y, Ono K, Nakatochi M, Ichihara S, Kabata R, et al. Significant association of RNF213 p.R4810K, a moyamoya susceptibility variant, with coronary artery disease. *PLoS One* 2017;**12**(4):e0175649.

125. Noda H, Maruyama K, Iso H, Dohi S, Terai T, Fujioka S, et al. Prediction of myocardial infarction using coronary risk scores among Japanese male workers: 3M Study. *J Atheroscler Thromb* 2010;**17**(5):452-9.

126. Oda E, Goto M, Matsushita H, Takarada K, Tomita M, Saito A, et al. The association between obesity and acute myocardial infarction is age- and gender-dependent in a Japanese population. *Heart Vessels* 2013;**28**(5):551-8.

127. Eguchi E, Iso H, Tanabe N, Yatsuya H, Tamakoshi A. Is the association between healthy lifestyle behaviors and cardiovascular mortality modified by overweight status? The Japan Collaborative Cohort Study. *Prev Med* 2014;**62**:142-7.

128. Matsunaga M, Yatsuya H, Iso H, Yamashita K, Li Y, Yamagishi K, et al. Similarities and differences between coronary heart disease and stroke in the associations with cardiovascular risk factors: The Japan Collaborative Cohort Study. *Atherosclerosis* 2017;**261**:124-30.
