# Supplementary material for: The relationship of cigarette smoking in Japan to lung cancer, COPD, ischemic heart disease and stroke: A systematic review
Source: F1000Res. 2018 Feb 19;7:204. [Version 1] doi: 10.12688/f1000research.14002.1 (PMC6367657; doi:10.12688/f1000research.14002.1)
Supplement: Supplementary file 3 [file f1000research-7-15218-s0002.tgz › 73c9c793-3ec8-414b-89ef-a8e4e3a21d12.docx]

**Supplementary File 2**

**Relative risks by amount smoked and duration of quitting**

Lung cancer

See Table S2-1 (amount smoked) and Table S2-2 (duration of quitting)

COPD

No data for amount smoked

Only one study for duration of quitting (see footnote to Table 6 of main paper)

IHD

See Table S2-3 (amount smoked) and Table S2-4 (duration of quitting)

Stroke

See Table S2-5 (amount smoked) and Table S206 (duration of quitting)

TABLE S2-1 Lung cancer relative risks for amount smoked (compared to never smokers)

| Study | Sex | Amount smoked (cigs/day) | RR (95% CI) |
| --- | --- | --- | --- |
|  |  |  |  |
| HITOSU | M | 1-14 | 2.08 (0.90-4.83) |
|  |  | 15-24 | 2.82 (1.25-6.36) |
|  |  | 25+ | 4.68 (1.97-11.11) |
|  | F | 1-14 | 3.11 (1.77-5.46) |
|  |  | 15+ | 3.17 (1.07-9.36) |
|  |  |  |  |
| SEGI2 | M | 1-9 | 2.10 (0.86-5.16) |
|  |  | 10-19 | 3.10 (1.40-6.84) |
|  |  | 20-29 | 3.40 (1.55-7.45) |
|  |  | 30-39 | 6.90 (2.78-17.14) |
|  |  | 40+ | 7.90 (3.40-18.37) |
|  | F | 1-9 | 2.90 (1.09-7.70) |
|  |  | 10-19 | 1.44 (0.61-3.40) |
|  |  | 20+ | 1.03 (0.34-3.15) |
|  |  |  |  |
| TSUGAN | M | 1-15 | 0.90 (0.36-2.28) |
|  |  | 16-35 | 1.22 (0.55-2.73) |
|  |  | 36+ | 1.66 (0.65-4.20) |
|  |  |  |  |
| HIRAYA | M | 1-9 | 2.06 (1.49-2.85) |
|  |  | 10-19 | 4.00 (3.20-4.99) |
|  |  | 20+ | 6.24 (5.07-7.68) |
|  |  | 1-9 | 2.25 (1.64-3.08) |
|  |  | 10-19 | 2.56 (1.85-3.54) |
|  |  | 20+ | 4.47 (2.73-7.33) |
|  |  |  |  |
| YAMAGU | M+F | 1-20 | 3.75 (1.89-7.47) |
|  |  | 21+ | 12.14 (5.10-28.90) |
|  |  |  |  |
| GAO2 | M | 1-19 | 3.36 (1.57-7.19) |
|  |  | 20-29 | 7.54 (3.71-15.30) |
|  |  | 30+ | 10.63 (5.08-22.22) |
|  |  |  |  |
| SOBUE | M | 1-19 | 3.52 (2.27-5.47) |
|  |  | 20-29 | 4.00 (2.63-6.09) |
|  |  | 30+ | 4.55 (2.97-6.96) |
|  |  |  |  |
| WAKAI | M | 1-19 | 1.80 (0.81-4.02) |
|  |  | 20-29 | 4.01 (1.91-8.41) |
|  |  | 30+ | 9.19 (4.20-20.10) |
|  |  |  |  |
| STELL2 | M | 1-19 | 2.60 (1.40-4.90) |
|  |  | 20-29 | 4.30 (2.40-7.60) |
|  |  | 30+ | 9.30 (5.20-16.70) |
|  |  |  |  |
| SOBUE3 | M | 1-19 | 4.18 (2.64-6.62) |
|  |  | 20-29 | 4.35 (2.83-6.68) |
|  |  | 30-39 | 3.78 (2.26-6.35) |
|  |  | 40+ | 3.77 (2.19-6.49) |
|  |  |  |  |
| KAWAMI | M | 1-20 | 5.99 (1.84-19.51) |
|  |  | 21-40 | 11.16 (3.31-37.66) |
|  |  | 41+ | 13.10 (2.88-59.70) |
|  | F | 1-20 | 3.40 (1.29-8.93) |
|  |  | 21-40 | 10.25 (1.19-88.26) |
|  |  |  |  |
| MARUG2 | M | 1-20 | 1.94 (1.31-2.87) |
|  |  | 21-39 | 3.38 (2.27-5.05) |
|  |  | 40-99 | 4.61 (2.80-7.57) |
|  | F | 1-20 | 1.98 (1.18-3.32) |
|  |  | 21+ | 4.37 (1.57-12.20) |
|  |  |  |  |
| JACC (OZASA) | M | 1-14 | 3.12 (2.25-4.33) |
|  |  | 15-24 | 5.12 (3.87-6.76) |
|  |  | 25+ | 7.37 (5.43-10.00) |
|  | F | 1-14 | 4.79 (3.13-7.31) |
|  |  | 15-24 | 4.03 (2.23-7.28) |
|  |  | 25+ | 3.95 (0.97-16.00) |
|  |  |  |  |
| AKIBA | M | 1-14 | 3.50 (2.20-6.00) |
|  |  | 15-24 | 6.10 (3.90-9.50) |
|  |  | 25+ | 9.10 (5.40-15.90) |
|  | F | 1-14 | 3.60 (2.60-5.00) |
|  |  | 15+ | 5.80 (3.30-9.50) |
|  |  |  |  |

TABLE S2-2 Lung cancer relative risks by time quit (compared to never smokers)

| Study | Sex | Years quit | RR (95% CI) |
| --- | --- | --- | --- |
|  |  |  |  |
| HIRAYA | M | 1-4 | 2.03 (1.10-3.75) |
|  |  | 5-9 | 1.59 (0.66-3.82) |
|  |  | 10+ | 1.38 (0.59-3.21) |
|  | F | 1-4 | 3.72 (1.12-12.37) |
|  |  | 5-9 | 3.29 (0.56-19.50) |
|  |  | 10+ | 0.97 (0.03-32.06) |
|  |  |  |  |
| GAO2 | M | 1-4 | 5.14 (2.31-11.40) |
|  |  | 5-9 | 3.48 (1.51-8.01) |
|  |  | 10-14 | 3.83 (1.55-9.46) |
|  |  | 15-19 | 3.35 (1.05-10.66) |
|  |  | 20+ | 1.38 (0.51-3.74) |
|  |  |  |  |
| SOBUE^a^ | M | 1-4 | 4.79 (2.98-7.71) |
|  |  | 5-9 | 3.16 (1.90-5.28) |
|  |  | 10-14 | 3.04 (1.68-5.49) |
|  |  | 15-19 | 3.36 (1.72-6.56) |
|  |  | 20-24 | 2.83 (1.32-6.09) |
|  |  | 25+ | 1.85 (0.92-3.71) |
|  |  |  |  |
| WAKAI | M | 5-9 | 2.48 (1.04-5.92) |
|  |  | 10-19 | 3.63 (1.56-8.44) |
|  |  | 20+ | 1.00 (0.35-2.83) |
|  |  |  |  |
| STELL2 | M | 1-4 | 5.67 (2.65-12.13) |
|  |  | 5-9 | 5.04 (2.54-9.99) |
|  |  | 10-15 | 1.26 (0.55-2.88) |
|  |  | 16+ | 1.26 (0.62-2.56) |
|  |  |  |  |
| JPHC (SOBUE) | M | <9 | 3.00 (1.90-5.00) |
|  |  | 10-19 | 1.80 (1.00-3.30) |
|  |  | 20+ | 1.00 (0.40-2.40) |
|  |  |  |  |
| JACC (OZASA) | M | <5 | 3.66 (2.58-5.19) |
|  |  | 5-14 | 2.19 (1.54-3.12) |
|  |  | 15+ | 1.17 (0.77-1.79) |
|  | F | <5 | 2.10 (0.52-8.50) |
|  |  | 5-14 | 3.13 (1.15-8.50) |
|  |  | 15+ | 2.15 (0.53-8.73) |
|  |  |  |  |
| OSAKI | M | <5 | 2.80 (1.10-7.10) |
|  |  | 5-9 | 3.90 (1.60-9.40) |
|  |  | 10-19 | 1.90 (0.80-4.60) |
|  |  | 20+ | 1.40 (0.60-3.60) |
|  |  |  |  |
|  |  |  |  |
| 3 STUDIES | M | 0-4 | 3.68 (2.79-4.86) |
|  |  | 5-9 | 2.52 (1.84-3.45) |
|  |  | 10-14 | 1.87 (1.29-2.71) |
|  |  | 15-19 | 1.24 (0.75-2.07) |
|  |  | 20-24 | 1.09 (0.60-1.99) |
|  |  | 25+ | 0.59 (0.32-1.11) |
|  |  |  |  |

^a^ Source is reference^1^

TABLE S2-3 IHD relative risks for amount smoked (compared to never smokers)

| Study | Sex | Age | Amount smoked (cigs/day) | RR (95% CI) |
| --- | --- | --- | --- | --- |
|  |  |  |  |  |
| HIRAYA | M | 40+ | 1-9 | 1.68 (1.40-2.03) |
|  |  |  | 10-19 | 1.63 (1.41-1.87) |
|  |  |  | 20+ | 1.95 (1.69-2.24) |
|  | F |  | 1-9 | 1.69 (1.38-2.08) |
|  |  |  | 10-19 | 2.25 (1.85-2.73) |
|  |  |  | 20+ | 3.77 (2.77-5.13) |
|  |  |  |  |  |
| 3 STUDIES | M | 40-79 | 1-9 | 1.62 (0.97-2.70) |
|  |  |  | 10-19 | 1.80 (1.21-2.68) |
|  |  |  | 20+ | 2.28 (1.56-3.34) |
|  | F |  | 1-9 | 1.95 (0.90-4.23) |
|  |  |  | 10-19 | 1.89 (0.90-3.98) |
|  |  |  | 20+ | 3.09 (1.44-6.63) |
|  |  |  |  |  |
| UESHIM | M | 30+ | 1-20 | 1.56 (0.54-4.53) |
|  |  |  | 21+ | 4.25 (1.42-12.80) |
|  | F^a^ |  | 1-20 | 1.27 (0.43-3.76) |
|  |  |  |  |  |
| YAMAGI | M | 40-69 | 1-20 | 4.10 (1.40-11.80) |
|  |  |  | 21+ | 4.60 (1.60-12.90) |
|  |  |  |  |  |
| JPHC (ESMAK) | M | 45-74 | 1-19 | 2.01 (1.50-2.67) |
|  |  |  | 20-29 | 2.37 (1.80-3.10) |
|  |  |  | 30+ | 2.56 (1.83-3.59) |
|  | F |  | 1-19 | 2.46 (1.52-4.62) |
|  |  |  | 20-29 | 3.01 (1.76-8.18) |
|  |  |  | 30+ | 3.99 (2.11-9.10) |
|  |  |  |  |  |
| KONDO | M | 20-61 | 1-10 | 6.80 (1.66-33.30) |
|  |  |  | 11-20 | 3.93 (1.29-17.00) |
|  |  |  | 21+ | 5.82 (1.80-25.90) |
|  |  |  |  |  |
| HATANA | M | 30-39 | 1-20 | 1.94 (0.93-4.07) |
|  |  |  | 21+ | 3.12 (1.21-8.06) |
|  | M | 40-55 | 1-20 | 1.15 (0.83-1.59) |
|  |  |  | 21+ | 1.81 (1.25-2.62) |
|  |  |  |  |  |

^a^ No cases for amount smoked 21+

TABLE S2-4 IHD relative risks by time quit (compared to never smokers)

| Study | Sex | Age | Years quit | RR (95% CI) |
| --- | --- | --- | --- | --- |
|  |  |  |  |  |
| HIRAYA | M | 40+ | 1-4 | 1.50 (1.03-2.20) |
|  |  |  | 5-9 | 1.73 (1.11-2.73) |
|  |  |  | 10+ | 0.94 (0.54-1.64) |
|  | F |  | 1-4 | 0.41 (0.05-3.37) |
|  |  |  | 5-9 | 1.19 (0.20-6.91) |
|  |  |  | 10+ | 0.91 (0.12-6.76) |
|  |  |  |  |  |
| 3 STUDIES | M | 40-79 | <2 | 1.97 (1.05-3.70) |
|  |  |  | 2-4 | 2.94 (1.90-4.56) |
|  |  |  | 5-9 | 2.83 (1.86-4.31) |
|  |  |  | 10-14 | 1.69 (1.06-2.71) |
|  |  |  | 15+ | 1.28 (0.82-1.98) |
|  | F |  | <2 | 2.79 (0.89-8.74) |
|  |  |  | 2-4 | 3.40 (1.47-7.84) |
|  |  |  | 5-9 | 1.46 (0.57-3.75) |
|  |  |  | 10-14 | 0.60 (0.13-2.77) |
|  |  |  | 15+ | 0.33 (0.07-1.54) |
|  |  |  |  |  |

TABLE S2-5 Stroke relative risks for amount smoked (compared to never smokers)

| Study | Sex | Age | Amount smoked (cigs/day) | RR (95% CI) |
| --- | --- | --- | --- | --- |
|  |  |  |  |  |
| HIRAYA | M | 40+ | 1-9 | 1.23 (1.12-1.34) |
|  |  |  | 10-19 | 1.07 (1.00-1.13) |
|  |  |  | 20+ | 0.96 (0.90-1.03) |
|  | F |  | 1-9 | 1.17 (1.05-1.30) |
|  |  |  | 10-19 | 1.09 (0.97-1.23) |
|  |  |  | 20+ | 1.34 (1.06-1.70) |
|  |  |  |  |  |
| 3 STUDIES | M | 40-79 | 1-9 | 1.96 (1.42-2.72) |
|  |  |  | 10-19 | 1.41 (1.07-1.85) |
|  |  |  | 20+ | 1.43 (1.09-1.86) |
|  | F |  | 1-9 | 2.13 (1.31-3.48) |
|  |  |  | 10-19 | 2.27 (1.43-3.61) |
|  |  |  | 20+ | 2.85 (1.72-4.70) |
|  |  |  |  |  |
| UESHIM | M | 30+ | 1-20 | 1.60 (0.91-2.79) |
|  |  |  | 21+ | 2.17 (1.09-4.30) |
|  | F |  | 1-20 | 1.42 (0.72-2.78) |
|  |  |  | 21+ | 3.91 (1.18-12.90) |
|  |  |  |  |  |
| YAMAGI | M | 40-69 | 1-20 | 1.10 (0.70-1.70) |
|  |  |  | 21+ | 1.60 (1.10-2.40) |
|  |  |  |  |  |
| KONDO | M | 20-61 | 1-10 | 0.67 (0.10-2.55) |
|  |  |  | 11-20 | 2.46 (1.26-5.25) |
|  |  |  | 21+ | 2.21 (0.97-5.19) |
|  |  |  |  |  |

TABLE S2-6 Stroke relative risks by time quit (compared to never smokers)

| Study | Sex | Age | Years quit | RR (95% CI) |
| --- | --- | --- | --- | --- |
|  |  |  |  |  |
| HIRAYA | M | 40+ | 1-4 | 1.22 (1.03-1.46) |
|  |  |  | 5-9 | 0.82 (0.63-1.08) |
|  |  |  | 10+ | 0.80 (0.62-1.03) |
|  | F |  | 1-4 | 1.50 (0.91-2.47) |
|  |  |  | 5-9 | 1.49 (0.75-2.95) |
|  |  |  | 10+ | 1.61 (0.84-3.10) |
|  |  |  |  |  |
| 3 STUDIES | M | 40-79 | <2 | 1.73 (1.14-2.62) |
|  |  |  | 2-4 | 1.54 (1.11-2.13) |
|  |  |  | 5-9 | 1.44 (1.06-1.97) |
|  |  |  | 10-14 | 1.03 (0.73-1.46) |
|  |  |  | 15+ | 0.95 (0.71-1.29) |
|  | F |  | <2 | 1.06 (0.38-2.92) |
|  |  |  | 2-4 | 1.06 (0.62-1.82) |
|  |  |  | 5-9 | 1.12 (0.84-1.49) |
|  |  |  | 10-14 | 0.75 (0.37-1.54) |
|  |  |  | 15+ | 1.00 (0.78-1.28) |
|  |  |  |  |  |

**Reference**

1. Sobue T, Suzuki T, Fujimoto I, Matsuda M, Doi O, Mori T, et al. Lung cancer risk among exsmokers. *Jpn J Cancer Res* 1991;**82**:273-9.
