# Supplementary material for: The relationship of cigarette smoking in Japan to lung cancer, COPD, ischemic heart disease and stroke: A systematic review
Source: F1000Res. 2018 Feb 19;7:204. [Version 1] doi: 10.12688/f1000research.14002.1 (PMC6367657; doi:10.12688/f1000research.14002.1)
Supplement: Supplementary file 4 [file f1000research-7-15218-s0003.tgz › 30bb7c15-bf32-43f8-b4e2-6e4f674b5577.docx]

**Supplementary File 3**

**More detailed meta-analysis results**

**for cardiovascular disease and lung cancer**

The following pages present more detailed meta-analysis tables, firstly for lung cancer (Table S3-1) and then for cardiovascular disease (Table S3-2). There were too little data for COPD to justify more detailed meta-analyses.

TABLE S3-1 Detailed meta-analysis results for lung cancer

| Characteristic^a^ | Level | Statistic^b^ | Current smoking | Ex-smoking |
| --- | --- | --- | --- | --- |
|  |  |  |  |  |
| All | All | n | 39 | 34 |
|  |  | R | 3.59 (3.25-3.96) | 2.26 (2.03-2.52) |
|  |  | H, P_H_ | 2.98, p<0.001 | 1.50, p<0.05 |
|  |  |  |  |  |
| Sex | Male | n | 20 | 18 |
|  |  | R | 4.20 (3.74-4.72) | 2.36 (2.12-2.63) |
|  |  |  |  |  |
|  | Female | n | 13 | 10 |
|  |  | R | 3.00 (2.61-3.44) | 2.35 (1.70-3.25) |
|  |  |  |  |  |
|  | Combined | n | 6 | 6 |
|  |  | R | 3.27 (2.51-4.28) | 2.04 (1.51-2.75) |
|  |  |  |  |  |
|  | Between levels | P_B_ | <0.001 | NS |
|  |  |  |  |  |
| Publication year | Before 1980 | n | 4 | 2 |
|  |  | R | 2.64 (1.86-3.74) | 5.03 (2.62-9.66) |
|  |  |  |  |  |
|  | 1980-1999 | n | 12 | 10 |
|  |  | R | 3.63 (3.02-4.36) | 2.29 (1.92-7.55) |
|  |  |  |  |  |
|  | 2000 or later | n | 23 | 22 |
|  |  | R | 3.68 (3.24-4.18) | 2.18 (1.93-2.47) |
|  |  |  |  |  |
|  | Between levels | P_B_ | NS | <0.05 |
|  |  |  |  |  |
| Number of cases | <250 | n | 9 | 8 |
|  |  | R | 3.40 (2.39-4.83) | 2.57 (1.89-3.45) |
|  |  |  |  |  |
|  | 250-499 | n | 11 | 9 |
|  |  | R | 3.72 (2.94-4.72) | 2.58 (2.16-3.09) |
|  |  |  |  |  |
|  | 500-999 | n | 8 | 8 |
|  |  | R | 3.63 (3.04-4.34) | 2.10 (1.67-2.65) |
|  |  |  |  |  |
|  | 1000+ | n | 11 | 9 |
|  |  | R | 3.59 (3.06-4.21) | 2.10 (1.80-2.44) |
|  |  |  |  |  |
|  | Between levels | P_B_ | NS | NS |
|  |  |  |  |  |
| Number of adjustment variables | 0 | n | 4 | 4 |
|  |  | R | 2.66 (1.80-3.94) | 1.97 (1.16-3.34) |
|  |  |  |  |  |
|  | 1 | n | 17 | 12 |
|  |  | R | 3.32 (2.84-3.87) | 2.46 (2.07-2.92) |
|  |  |  |  |  |
|  | 2 or more | n | 18 | 18 |
|  |  | R | 4.06 (3.61-4.58) | 2.20 (1.98-2.45) |
|  |  |  |  |  |
|  | Between levels | P_B_ | <0.05 | NS |
|  |  |  |  |  |
| Study type | Case-control | n | 24 | 20 |
|  |  | R | 3.37 (2.92-3.87) | 2.32 (1.94-2.77) |
|  |  |  |  |  |
|  | Prospective | n | 15 | 14 |
|  |  | R | 3.89 (3.37-4.49) | 2.24 (1.99-2.53) |
|  |  |  |  |  |
|  | Between levels | P_B_ | NS | NS |
|  |  |  |  |  |

^a^ Results (not shown) were also similar by definition of current smoking (cigarettes or any product) or its denominator (never cigarettes

or never any products)

^b^ Footnote as in Table 3 of main paper

TABLE S3-2 Detailed meta-analysis results for cardiovascular diseases

|  |  |  | IHD |  |  | Stroke |  |
| --- | --- | --- | --- | --- | --- | --- | --- |
| Characteristic | Level | Statistic^a^ | Current smoking | Ex-smoking |  | Current smoking | Ex-smoking |
|  |  |  |  |  |  |  |  |
| All | All | n | 20 | 17 |  | 16 | 16 |
|  |  | R | 2.21 (1.96-2.50) | 1.46 (1.24-1.71) |  | 1.40 (1.25-1.57) | 1.05 (0.96-1.15) |
|  |  | H_,_ P_H_ | 2.53, p<0.001 | 1.58, <0.1 |  | 5.21, p<0.001 | 1.26, NS |
|  |  |  |  |  |  |  |  |
| Sex | Male | n | 12 | 10 |  | 9 | 9 |
|  |  | R | 1.98 (1.74-2.25) | 1.37 (1.18-1.61) |  | 1.32 (1.16-1.51) | 0.98 (0.91-1.06) |
|  |  |  |  |  |  |  |  |
|  | Female | n | 8 | 7 |  | 7 | 7 |
|  |  | R | 2.59 (2.06-3.27) | 1.75 (1.17-2.60) |  | 1.50 (1.16-1.94) | 1.29 (1.06-1.55) |
|  |  |  |  |  |  |  |  |
|  | Between levels | P_B_ | <0.05 | <0.1 |  | <0.01 | <0.01 |
|  |  |  |  |  |  |  |  |
| Age | <65 | n | 7 | 5 |  | 5 | 5 |
|  |  | R | 2.58 (1.74-3.84) | 1.75 (1.10-2.79) |  | 2.06 (1.43-2.97) | 1.27 (0.91-1.77) |
|  |  |  |  |  |  |  |  |
|  | 65+ | n | 4 | 4 |  | 4 | 4 |
|  |  | R | 2.12 (1.78-3.53) | 1.70 (1.32-2.20) |  | 1.20 (1.07-1.35) | 1.04 (0.91-1.20) |
|  |  |  |  |  |  |  |  |
|  | All | n | 9 | 8 |  | 7 | 7 |
|  |  | R | 2.06 (1.82-2.33) | 1.25 (1.08-1.44) |  | 1.20 (1.10-1.30) | 1.03 (0.89-1.20) |
|  |  |  |  |  |  |  |  |
|  | Between levels | P_B_ | NS | <0.05 |  | <0.001 | NS |
|  |  |  |  |  |  |  |  |
| Publication year | Before 2005 | n | 5 | 5 |  | 5 | 5 |
|  |  | R | 1.82 (1.65-2.02) | 1.38 (0.93-2.04) |  | 1.17 (1.06-1.29) | 1.14 (0.88-1.49) |
|  |  |  |  |  |  |  |  |
|  | 2005 on | n | 15 | 12 |  | 11 | 11 |
|  |  | R | 2.33 (2.01-2.70) | 1.48 (1.22-1.79) |  | 1.50 (1.26-1.78) | 1.01 (0.92-1.11) |
|  |  |  |  |  |  |  |  |
|  | Between levels | P_B_ | <0.01 | NS |  | <0.001 | NS |
|  |  |  |  |  |  |  |  |
| Number of cases | <250 | n | 6 | 4 |  | 3 | 3 |
|  |  | R | 2.07 (1.32-3.26) | 1.54 (0.70-3.40) |  | 1.79 (1.26-2.54) | 1.34 (0.86-2.09) |
|  |  |  |  |  |  |  |  |
|  | 251-999 | n | 10 | 9 |  | 5 | 5 |
|  |  | R | 2.51 (2.14-2.93) | 1.60 (1.25-2.05) |  | 1.52 (1.20-1.93) | 1.06 (0.84-1.33) |
|  |  |  |  |  |  |  |  |
|  | 1000+ | n | 4 | 4 |  | 8 | 8 |
|  |  | R | 1.92 (1.71-2.17) | 1.29 (1.09-1.53) |  | 1.32 (1.16-1.51) | 1.04 (0.93-1.17) |
|  |  |  |  |  |  |  |  |
|  | Between levels | P_B_ | <0.001 | NS |  | <0.01 | NS |
|  |  |  |  |  |  |  |  |
| Adjustment variables other than age | 0-5 | n | 13 | 13 |  | 13 | 13 |
|  |  | R | 2.27 (1.93-2.67) | 1.59 (1.33-1.90) |  | 1.44 (1.25-1.66) | 1.10 (0.99-1.22) |
|  |  |  |  |  |  |  |  |
|  |  |  |  |  |  |  |  |
|  | 6+ | n | 7 | 4 |  | 3 | 3 |
|  |  | R | 2.14 (1.74-2.64) | 1.24 (0.96-1.59) |  | 1.28 (1.14-1.43) | 0.91 (0.79-1.04) |
|  |  |  |  |  |  |  |  |
|  | Between levels | P_B_ | NS | <0.05 |  | NS | <0.05 |
|  |  |  |  |  |  |  |  |

^a^ Footnote as in Table 3 of main paper
