# Supplementary material for: The relationship of cigarette smoking in Japan to lung cancer, COPD, ischemic heart disease and stroke: A systematic review
Source: F1000Res. 2018 Feb 19;7:204. [Version 1] doi: 10.12688/f1000research.14002.1 (PMC6367657; doi:10.12688/f1000research.14002.1)
Supplement: Peer review history

# Peer review: Referee response for version 1

**DOI:** 10.5256/f1000research.15218.r38476

**Published:** 2019-02-06

**Authors:** Wolf-Dieter Heller

**Affiliations:** 1 Institute of Economics, Econometrics and Statistics, Karlsruhe Institute of Technology, Karlsruhe, Germany

The authors report the results of meta-analyses based on studies in Japan relating cigarette smoking to the risk of the four major smoking-related diseases. Data are summarized for current and former smokers and in relation to amount smoked and time quit. The relative risks reported for Japan are compared with those for other Asian and Western studies reported in published meta-analyses.

 The results shown are mostly quite similar to those reported elsewhere, with relative risks for current smoking elevated for all four diseases, relative risks for former smoking somewhat lower, and evidence of a dose response with increasing amount smoked and decreasing time quit. While current smoking relative risks for heart disease and stroke for Japan are similar to those in other countries, it is notable that, for lung cancer, the relative risks for Japan are much lower than seen in Western countries, though similar to those seen in other Asian countries. The paper includes an interesting discussion about the reasons why this might be so.

 Generally, the paper is very well structured and clearly written, and the material presented provides considerable reassurance about the completeness of the searches and the accuracy of the meta-analysis results presented. Over the last decades Peter Lee and his co-authors have published a broad range of papers dealing with the risk of smoking in general. Monitoring these publications over many years I was always impressed how clearly the papers are written, especially the MA ones.

 The results of the present publication are convincing, clear and concise. Concerning the lung cancer results this paper again points towards an important aspect in public health which definitely needs further research: the substantially lower RR in Japan compared to western countries.

I have read this submission. I believe that I have an appropriate level of expertise to confirm that it is of an acceptable scientific standard.

# Peer review: Referee response for version 1

**DOI:** 10.5256/f1000research.15218.r31116

**Published:** 2018-03-16

**Authors:** Joshua E. Muscat

**Affiliations:** 1 Department of Public Health Sciences, Penn State Tobacco Center of Regulatory Science, Penn State Health Milton S. Hershey Medical Center, Hershey, PA, USA

These authors have vast experience in MA and the analysis is carefully done and interpreted. The methods conform to appropriate MA methodology.

 This is a useful paper as differences in smoking-related risks between Japan and Western countries have important implications with regard to potential cigarette toxicity or risk moderators. The conclusions are quite reasonable.

I have read this submission. I believe that I have an appropriate level of expertise to confirm that it is of an acceptable scientific standard.

# Response

**Published:** 2018-03-16

**Authors:** Peter Lee

**Affiliations:** P.N.Lee Statistics and Computing Ltd, UK

I thank Dr Muscat for his kind comments.
